# Selective Bidentate Coordination Reconstructs Residual PbI_2_ to Homogenize Interfacial Energetics in Perovskite Solar Cells

**DOI:** 10.1021/jacs.6c05316

**Published:** 2026-07-08

**Authors:** Yuanhao Tang, Chenjian Lin, Zhichen Nian, Thomas W. Gries, Jeong Hui Kim, Kevin R. Pedersen, Yu-Ting Yang, Siddha Hill, Vignesh Sathyaseelan, Yunfei Wang, Hanjun Yang, Han Zhao, Wenzhan Xu, Zheng-Fei Liu, Xiyu Luo, Yanyan Li, Chongli Yuan, Libai Huang, Chenhui Zhu, Kenneth R. Graham, Artem Musiienko, Brett M. Savoie, Letian Dou

**Affiliations:** † Davidson School of Chemical Engineering, 311308 Purdue University, West Lafayette, Indiana 47907, United States; ‡ Department of Chemistry, 1371 Emory University, Atlanta, Georgia 30322, United States; § Chemical and Biomolecular Engineering, 6111 University of Notre Dame, South Bend, Indiana 46556, United States; ∥ Robotized Optoelectronic Material and Photovoltaic Engineering, 28340 Helmholtz-Zentrum Berlin für Materialien und Energie (HZB), Berlin 12489, Germany; ⊥ Department of Chemistry, 4530 University of Kentucky, Lexington, Kentucky 40506, United States; # Advanced Light Source, 1666 Lawrence Berkeley National Laboratory, Berkeley, California 94720, United States; ∇ Department of Chemistry, Purdue University, West Lafayette, Indiana 47907, United States

## Abstract

Spatially heterogeneous interfacial energetics, often originating from residual lead iodide (PbI_2_), represent a fundamental bottleneck to both the efficiency and operational stability of perovskite photovoltaics. Conventional PbI_2_ passivation strategies based on monodentate ligands or highly polar solvents either interact weakly with PbI_2_ or undesirably perturb the underlying three-dimensional perovskite lattice. Here, we report a diammonium bidentate strategy that selectively reconstructs residual PbI_2_ into corner-sharing PbI_6_ octahedra while preserving the bulk perovskite. Enabled by dual-site coordination, a newly designed thiophene-based bidentate ligand (MeXT) stabilizes PbI_6_ units during passivation and establishes spatially homogeneous interfacial energetics. Perovskite solar cells incorporating MeXT achieve 26.19% power conversion efficiency and retain over 80% of their initial efficiency after 1000 h of continuous 1-sun illumination at 75 °C. This dual-anchor coordination passivation strategy establishes a general design principle for selectively treating residual PbI_2_ and creating electronically coherent and operationally stable interfaces in perovskite photovoltaics.

## Graphical abstract

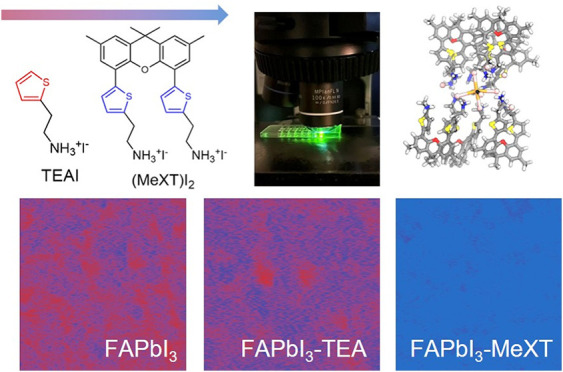

## Introduction

The rapid rise in power conversion efficiency (PCE) of metal halide perovskite solar cells (PSCs) has brought them to the forefront of next-generation photovoltaic technologies.^1–5^ Yet long-term operational stability, particularly under simultaneous heat, light, or bias stress, still lags far behind industrial benchmarks.^6–9^ Device failure is often associated with spatially heterogeneous interfacial energetics, manifested as localized potential fluctuations and nonradiative recombination.^10–12^ Accordingly, rather than additive engineering aimed at improving intrinsic bulk stability, interface engineering, including surface defect passivation and the construction of two-dimensional/three-dimensional (2D/3D) heterostructures, has emerged as an integral approach to enhancing device robustness.^1–4,13–20^

A recurring motif in these strategies is the presence of residual PbI_2_ that segregates to grain boundaries during crystallization.^14,15,21–23^ Initial studies demonstrated that incorporating a moderate excess of lead iodide (PbI_2_) into the perovskite precursor solution can enhance film crystallinity, promote larger perovskite grain sizes, and improve film ambient stability.^24,25^ Moreover, the presence of surface-enriched PbI_2_ can facilitate post reactions with bulky organic ligands to form 2D perovskite, thereby enabling 2D/3D heterojunctions that promote energy level alignment, suppress interfacial nonradiative recombination, and improve device stability.^13–15,21^ These benefits have led to the widespread use of PbI_2_-rich precursor formulations.^1,3–5,21,23,26^ However, increasing evidence suggests that excess PbI_2_ often becomes spatially heterogeneous and introduces strong tensile stresses, leading to the lattice distortion.^27,28^ More importantly, the photodegradation of PbI_2_ triggers the formation of localized photophysical loss channels, ultimately compromising photostability under continuous illumination.^29–31^

One of the most widely used strategies to modulate residual surface PbI_2_ is to chemically convert it into more thermodynamically or electronically favorable phases.^32,33^ Such post-treatment protocols typically rely on treatments with bulky ammonium ligands in solvents such as isopropanol (IPA), dimethylformamide (DMF), or dimethyl sulfoxide (DMSO), which are capable of dissolving PbI_2_.^13,15,21,34,35^ While these methods can effectively reduce surface PbI_2_ and promote 2D layer formation, they often risk perturbing the underlying 3D perovskite lattice.^21,34^ Achieving uniform and well-defined 2D capping layers in such systems requires careful control of ligand solubility, solvent-perovskite interactions, and reaction kinetics. Furthermore, post-treatment with highly polar solvents may raise compatibility concerns for large-area device processing and module-scale applications.^36,37^ More critically, these approaches frequently fail to establish spatially uniform interfacial energetics across the entire perovskite film.

Here, we introduce a rational ligand design strategy employing bidentate ligands as coordination-engineered alternatives to conventional monodentate analogues. These bidentate ligands exhibit highly selective interaction with PbI_2_, enabling its targeted reconstruction while preserving the bulk perovskite lattice. This selectivity arises from bidentate coordination, which suppresses PbI_2_ aggregation and stabilizes dispersed Pb–I motifs through the formation of PbI_6_ octahedral units, thereby enabling controlled interfacial reconstruction of residual PbI_2_ under mild conditions. This approach yields perovskite films with homogeneous surface energetics and enhanced optoelectronic properties. Devices passivated by thiophene-based bidentate ligands exhibit a champion PCE of 26.19%, with negligible efficiency loss after 1000 h of continuous 1-sun illumination. Under accelerated aging conditions at 75 °C and 1-sun illumination, the devices retain over 80% of their initial efficiency after 1000 h, representing a benchmark for the stability of n-i-p-structured perovskite photovoltaics.

## Results and Discussion

### Molecular Design Enabling Selective PbI_2_ Reconstruction by Bidentate Ligands

To elucidate the selective interaction of bidentate ligands with PbI_2_, we synthesized the bidentate ligand MeX and compared its performance with the widely used monodentate ligand phenylethylammonium iodide (PEA). Leveraging the stronger Lewis acid–base interaction between sulfur and uncoordinated Pb^2+^, we further introduced a new thiophene-based bidentate ligand (MeXT) along with its monodentate analogue, 2-thiopheneethylamine iodide (TEA), to broaden the generality of our strategy (Figure 1a and Supporting Figures 1–3). Electrostatic potential (ESP) mapping confirmed a notably higher localized negative charge density near sulfur atoms within thiophene units compared to benzene, enhancing their affinity toward Pb^2+^ sites (Figure 1a). Density functional theory (DFT) calculations showed that bidentate ligand binds significantly stronger to PbI_2_ than monodentate ligand (Figure 1b and Supporting Figures 4–6). This energetic preference provides a driving force for selective interfacial enrichment of bidentate ligand at PbI_2_-rich interfaces during solution processing, reducing its propensity to detachment under processing and making it more likely to remain anchored at the inorganic surface. At the structural level, this interaction manifests as an intralayer dual-site anchoring motif, as revealed by both DFT calculations and single-crystal analysis, in which the two ammonium groups simultaneously interact with the PbX_6_ (X = I or Br) framework (Supporting Figures 7, 8, and Table S1). Such a configuration gives rise to a chelation-like, double-point interaction that stabilizes the local Pb coordination environment and underpins the selective reconstruction of residual PbI_2_.

**Figure 1:**
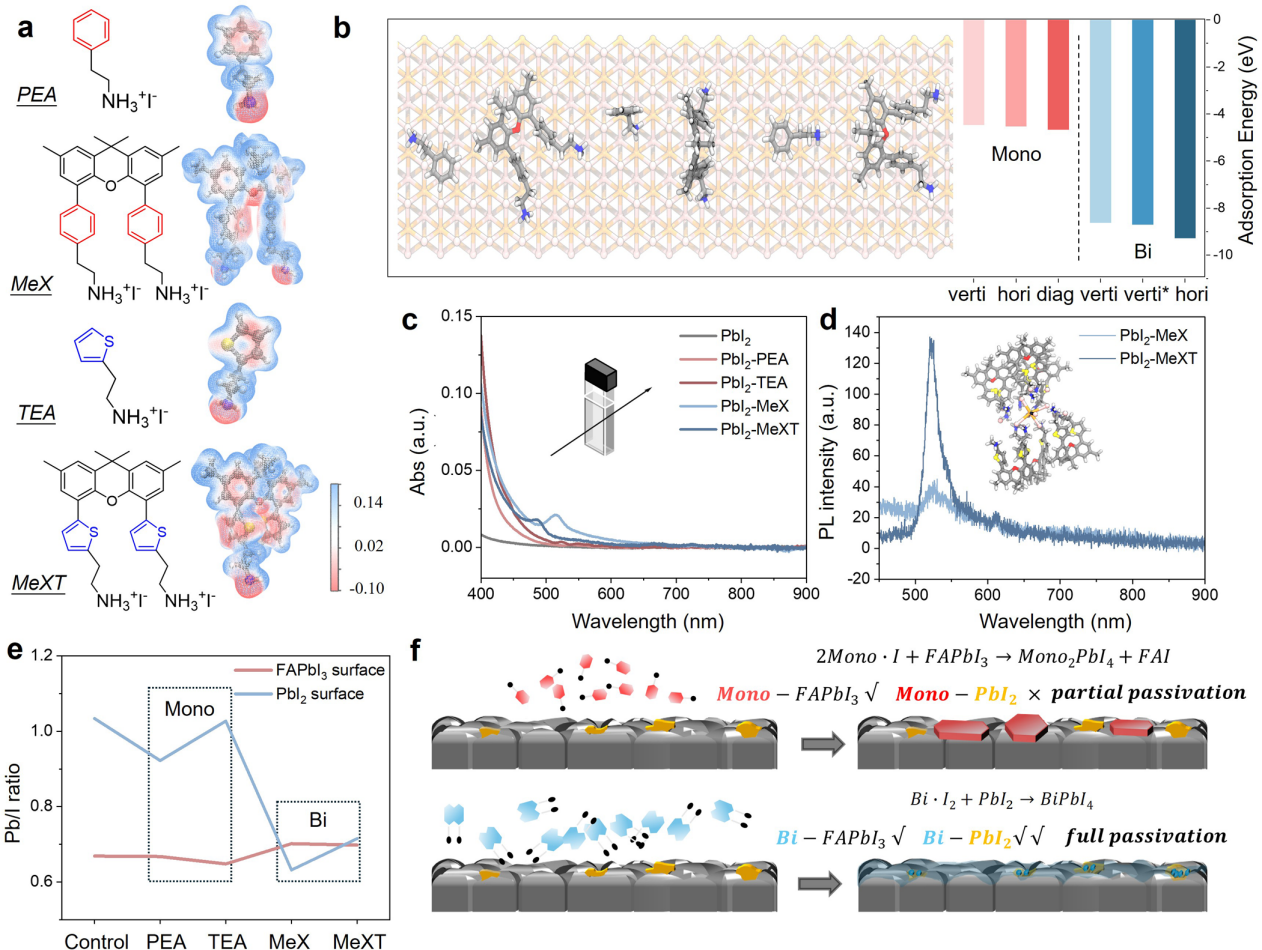
Molecular design and passivation mechanism of mono- and bidentate ligands. (a) Molecular structures and electrostatic potential maps of ligands. (b) Schematic images and simulated adsorption energies of PEA and MeX on PbI_2_ surfaces. UV–vis absorption spectra (c) and PL spectra (d) measured by dissolving PbI_2_ and ligands into a mixed solvent (CB/IPA; v/v = 9:1). The inset of (d) is the schematic image of ligand-encapsulated PbI_6_ unit in mixed solvent (CB/IPA; v/v = 9:1). (e) Surface Pb/I ratio of treated films determined by XPS. (f) Schematic showing different passivation routes. Color scheme for atomic representations used throughout: hydrogen (white), carbon (black), nitrogen (blue), oxygen (red), sulfur (yellow), iodine (light pink), and lead (orange).

Besides their stronger binding affinity, the pronounced interaction between bidentate ligands and PbI_2_ likely stems from their capacity to transform PbI_2_ into structurally stable PbI_6_ octahedra via robust coordination, facilitating efficient PbI_2_ dispersion and interfacial reconstruction. Liquid-phase ultraviolet–visible (UV–vis) spectroscopy supports this ligand-induced structural reconstruction. After filtration, pristine PbI_2_ dispersed in the mixture of chlorobenzene (CB) and isopropyl alcohol (IPA) (v/v = 9:1) yields an almost featureless spectrum (Figure 1c), consistent with the negligible solubility of PbI_2_ in noncoordinating solvents. The introduction of monodentate ligands PEA and TEA primarily enhanced the iodide-related absorption, with little evidence for the formation of discrete Pb–I coordination complexes. In contrast, solutions containing the bidentate ligands MeX and MeXT display pronounced excitonic absorption characteristic of [PbI_6_]^4–^ complexes at 513 and 485 nm, respectively. Control measurements on unfiltered PbI_2_ dispersions in the mixture of CB and IPA show a feature around 480 nm with a side peak at 575 nm, which may be from the scattering of suspended 2H hexagonal PbI_2_ crystallites. Direct dissolution of PbI_2_ in dimethylformamide (DMF) exhibits a wide absorption feature before 480 nm, which can be ascribed to [PbI_*x*_]^(*x*‑2)‑^ species (Supporting Figure 9).^38,39^ Additionally, liquid-phase photoluminescence (PL) exhibits a typical excitonic feature of 2D perovskite at 520 nm, further confirming the conversion and stabilization of corner-sharing PbI_6_ after incorporating bidentate ligands (Figure 1d).^40^ Owing to its stronger dual-site coordination with Pb^2+^, MeXT promotes the formation of a much more dispersed [PbI_6_]^4–^ species than MeX, leading to substantially brighter fluorescence (Supporting Figure 10). Solution-state nuclear magnetic resonance (NMR) spectroscopy in IPA-*d*_8_ further supports stronger ligand-PbI_2_ interactions for bidentate ligands. In contrast to the negligible shifts observed for PEA and TEA, MeX and MeXT show more pronounced chemical shift changes in the nonexchangeable protons (Supporting Figures 11, 12).

To further examine potential reconstruction and ligand-assisted passivation pathways, we performed molecular dynamics simulations using a machine-learning interatomic potential (MLIP). Although MLIP-molecular dynamics can, in principle, deliver near-DFT-accuracy dynamics at extended time and length scales and show the PbI_6_ stabilization effect of bidentate ligands, the trajectories for the present system exhibit unphysical artifacts that preclude mechanistic interpretation (Supporting Methods and Figures 13,14). Surface compositional analysis via X-ray photoelectron spectroscopy (XPS) corroborated these findings. We note that XPS-derived Pb/I ratios are semiquantitative and reflect the near-surface composition, which can deviate from the bulk stoichiometry due to surface termination effects and the facile loss of volatile iodide species under vacuum.^41^ While Pb/I ratios of monodentate-treated PbI_2_ films closely matched those of untreated PbI_2_, bidentate ligand treatment markedly decreased Pb/I ratios to levels comparable to pristine and passivated FAPbI_3_.^42^ This indicates a profound surface reconstruction, wherein layered PbI_2_ is effectively reorganized into stable, perovskite-like PbI_6_ octahedral frameworks through bidentate ligand coordination (Figures 1e,Supporting Figures 15–18, and Table S2). Accordingly, divergent passivation mechanisms were proposed (Figure 1f). Monodentate ligands, characterized by weaker PbI_2_ absorption and binding, may preferentially accumulate at A-site-terminated surface regions and aggregate into discrete 2D nanosheets, thus offering limited passivation of residual PbI_2_ and undercoordinated Pb^2+^ sites. In contrast, bidentate ligands with lower absorption energy exhibit robust binding affinity toward PbI_2_, enabling direct surface coordination and effective passivation of exposed Pb^2+^ sites and residual PbI_2_.

### Bidentate Ligands Enable Spatially Uniform Coordination and Interfacial Passivation of PbI_2_

We probe the coordination behavior of ligands on PbI_2_ by soaking bare PbI_2_ films, followed by spin coating. (Figure 2a). Optical microscopy (OM) with a 375 nm excitation source revealed that monodentate ligands produced a stark contrast in green fluorescence between the film edge and center, indicating highly localized 2D perovskite formation and nonuniform ligand distribution (Figure 2b).^43,44^ In contrast, bidentate ligands yielded uniform fluorescence across the entire film, suggesting more homogeneous interaction with the PbI_2_ surface (Figure 2b and Supporting Figure 19 and Movie S1–S4). The PL imaging further confirmed that bidentate ligand-treated films displayed suppressed and spatially uniform PL signals, whereas PEA and TEA treatments resulted in patchy, bright domains, pointing to aggregated coordination on the edge of the films (Figure 2d). To quantify this spatial heterogeneity further, we performed position-dependent PL mapping from the film center to the edge (Supporting Figures 20–25). For monodentate ligands, the PL intensity increases progressively toward the edge, accompanied by the emergence of larger and brighter emissive domains, highlighting strong spatial inhomogeneity (Supporting Figures 22, 23). In contrast, bidentate ligands display a much more uniform PL intensity distribution across the film (Supporting Figures 24,25). MeXT exhibits nearly invariant PL intensity from center to edge, indicating the most homogeneous ligand distribution (Supporting Figure 25). Consistent with the PL mapping, scanning electron microscopy (SEM) reveals distinct ligand-PbI_2_ interaction behaviors (Supporting Figure 26). Monodentate ligands show edge-localized platelet growth with negligible change at the center, whereas bidentate ligands induce uniform surface transformation. Notably, MeXT leads to aggregated nanostructures at the central, suggesting stronger interaction and surface reconstruction (Supporting Figure 27). The X-ray diffraction (XRD) analysis of the film center supported that monodentate ligands failed to induce the formation of 2D perovskite. While MeX and MeXT treatments led to the emergence of 2D diffraction peaks, confirming the structural modulation of surface PbI_2_ (Supporting Figures 28, 29).

**Figure 2:**
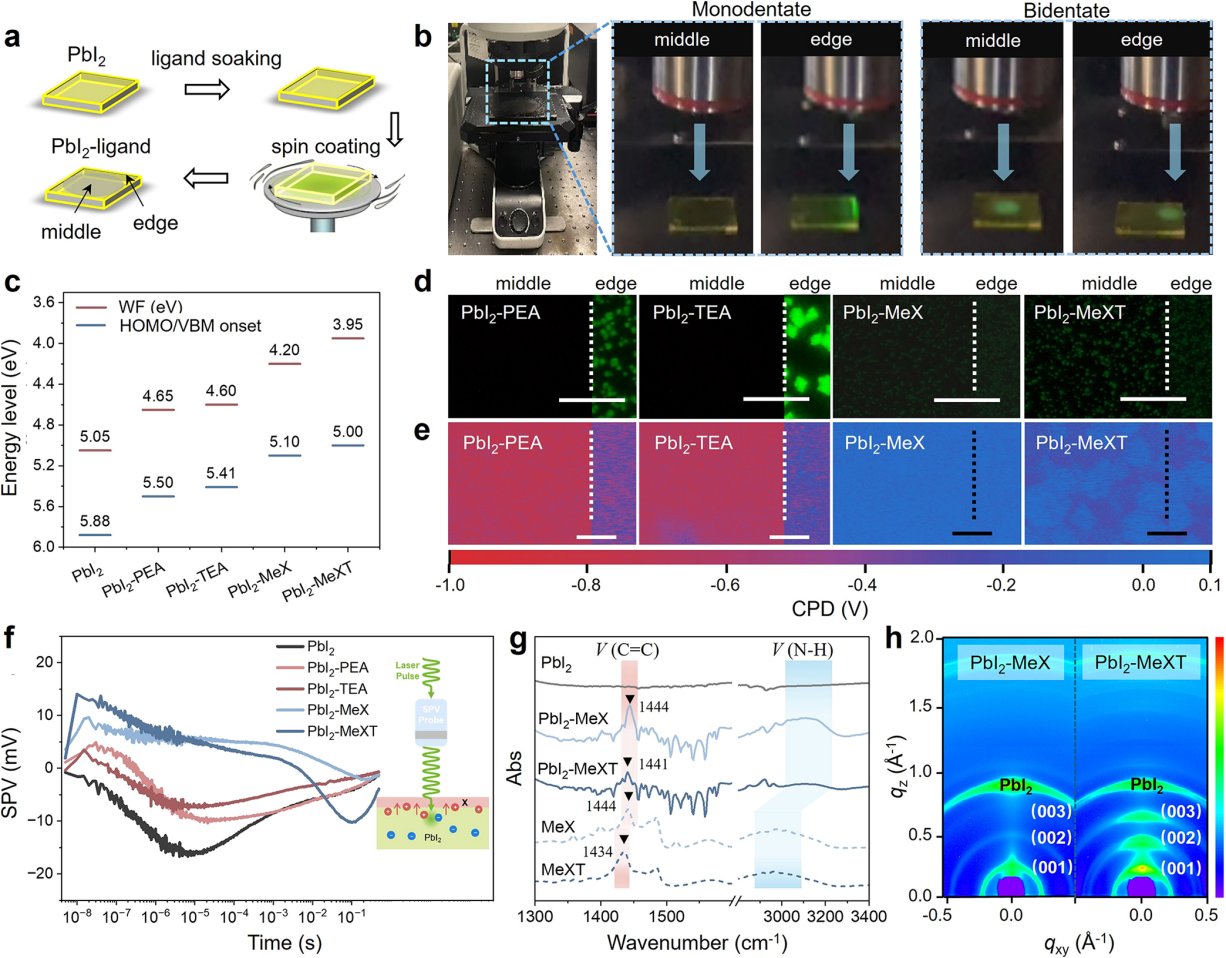
Strong and spatially uniform PbI_2_ coordination enabled by bidentate ligands. (a) Schematic of ligand soaking and spin coating. (b) OM images showing 2D fluorescence contrast at the middle vs the edge. (c) Energy-level alignment of ligand-treated films extracted from UPS, including WF and Highest Occupied Molecular Orbital (HOMO)/Valence Band Maximum (VBM) onset. (d) Fluorescence images of ligand-treated PbI_2_ films. (e, f) CPD maps from KPFM (e) and TRSPV transients (f) for ligand-treated PbI_2_ films. The inset in (f) illustrates the SPV probing configuration at the ligand-PbI_2_ interface. (g) FTIR spectra of ligand-PbI_2_ films. (h) GIWAXS 2D patterns of PbI_2_-MeX and PbI_2_-MeXT films. Scale bars: 20 μm (panel d) and 2 μm (panel e).

To map the spatial variation in electronic properties, Kelvin probe force microscopy (KPFM) was performed. The bidentate ligands produce potential difference (CPD) mappings that are spatially more uniform across the entire film, with minimal variation between the central region and the edges (Figure 2e). The monodentate-ligand-treated films display pronounced potential difference between the central region and the edges, together with excessive edge-localized hot spots, consistent with incomplete surface coverage and residual ionic heterogeneities (Figure 2e). Quantitatively, bidentate ligands raise the contact potential by around 0.7 V across the entire film (i.e, they decrease the work function), a shift that we ascribe to complete trap neutralization together with the formation of a coherent interfacial dipole (Supporting Figure 30).^45,46^ By contrast, monodentate ligands leave the CPD in the middle of the film essentially unchanged and produce only a modest, around 0.35 V positive shift at the edge. Ultraviolet photoelectron spectroscopy (UPS) further corroborates the trend, where bidentate ligands decrease the surface work function (WF) roughly twice as much as the monodentate analogues, making the film surface more n-type (Figure 2c,Supporting Figures 31, 32, and Table S3). Transient surface photovoltage (TRSPV) measurements were used to gain insight into the dynamics of charge extraction and interfacial charge trapping^47,48^ in PbI_2_ thin films. The polarity of the signal is determined by the type of charge carrier that migrates toward the SPV probe after excitation of the semiconducting material (Figure 2f, inset). Pristine PbI_2_ exhibits a pronounced negative SPV (Figure 2f), indicating dominant electron trapping by electron-accepting surface defects,^49^ e.g., uncoordinated Pb^2+^. Upon ligand coordination, this negative response is fully suppressed and replaced by a positive SPV, indicating effective passivation of electron-trapped states and a reversal of the interfacial photovoltage polarity. While both mono- and bidentate ligands mitigate electron trapping, bidentate ligands produce a substantially larger positive SPV amplitude in the ns range, indicating complete defect neutralization, with MeXT notably yielding the strongest positive response. Such neutralization of interfacial electron trap states by bidentate ligands would suppress interface nonradiative recombination and enhance charge extraction, thereby increasing *V*_oc_ in complete devices.

Fourier-transform infrared (FTIR) revealed distinct vibrational signatures for the ammonium ν­(N–H) and aromatic ν­(C=C) modes upon bidentate ligand binding (Supporting Figure 33).^50,51^ MeXT, which features two thiophene groups, exhibited a larger ν­(C=C) shift than its benzene analogue MeX. This enhanced perturbation likely originates from extended electronic coupling facilitated by the interaction between the thiophene sulfur and uncoordinated Pb^2+^ sites, thereby strengthening the overall binding (Figure 2g).^52^ Grazing-incidence wide-angle X-ray scattering (GIWAXS) measurements confirmed a notable enhancement of 2D diffraction features relative to bidentate ligands, especially MeXT, reflecting the highest degree of structural reordering and more extensive reorganization of surface PbI_2_ (Figure 2h). In contrast, monodentate ligands produced only weak 2D signatures mainly confined to the out-of-plane direction, suggesting limited and directionally selective interactions with the (00*l*) facets of PbI_2_ lattice (Supporting Figure 34).

### Bidentate Ligands Enable Selective and Spatially Uniform Passivation of Surface PbI_2_ in FAPbI_3_

Motivated by the strong affinity of bidentate ligands toward PbI_2_, we examined their passivation capability directly on perovskite surfaces. To highlight their selective action on residual PbI_2_, we introduced 10% excess of PbI_2_ into the FAPbI_3_ precursor solution. Scanning electron microscopy (SEM) revealed a bright contrast of PbI_2_ domains arising from distinct local surface electronic environments compared to the surrounding perovskite grains (Figure 3a).^53–55^ Upon passivation with monodentate ligands, residual PbI_2_ on the perovskite surface remained largely unchanged. KPFM images further confirmed the persistence of low-CPD PbI_2_ regions (Figure 3b). Simultaneously, large 2D perovskite nanosheets formed across the film surface, accompanied by underlying etching and morphological degradation where the nanosheets were established (the inset of Figure 3a). This structural erosion is expected to introduce additional defect states and compromise overall film stability (Supporting Figure 35).

**Figure 3:**
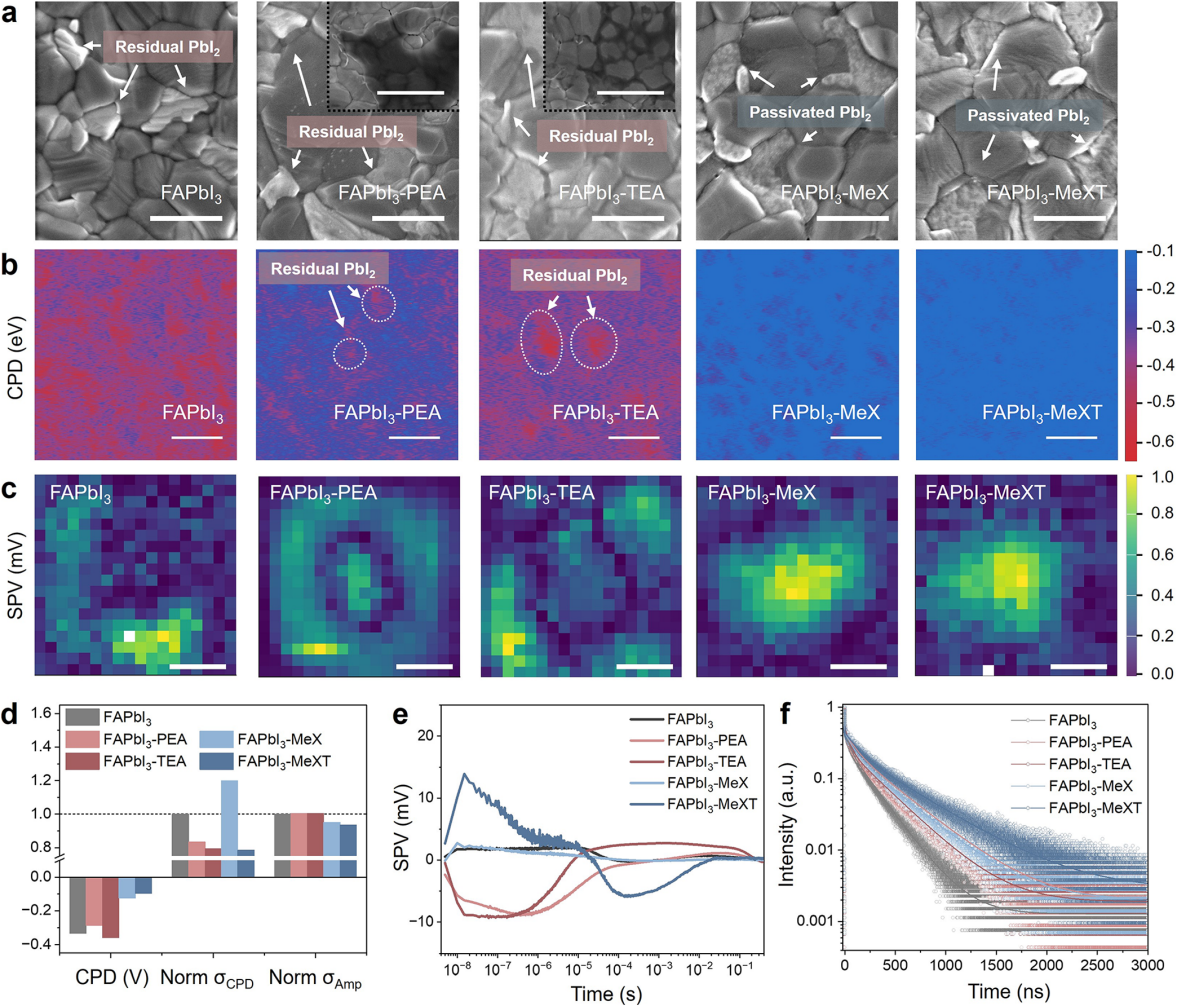
Selective passivation of surface PbI_2_ on FAPbI_3_ by bidentate ligands. (a–c) Top-view SEM images (a), KPFM contact potential difference (CPD) maps (b), and SPV spatial maps (c) of pristine and ligand-treated FAPbI_3_ films. In panel (a), the insets highlight representative regions where large plate-like crystallites are observed atop the underlying 3D FAPbI_3_ grains in monodentate-treated films. The CPD maps in (b) display the surface potential distribution (mV), while the SPV maps in (c) show the normalized photoinduced surface potential amplitude (a.u.), where brighter regions correspond to stronger local photoresponse. (d) Quantified CPD, CPD variation, and amplitude variation of ligand-passivated FAPbI_3_ films. (e) TRSPV transients of ligand-passivated FAPbI_3_ films. (f) TRPL measurements of ligand-passivated FAPbI_3_ films. Scale bars: 2 μm (panels a and b); 1 μm (the inset of panel a); 5 mm (panel c).

In sharp contrast, surfaces treated with bidentate ligands exhibited effective structural reorganization and selective reconstruction of residual PbI_2_ without the formation of large area 2D nanosheets (Figure 3a andSupporting Figure 36). AFM measurements show an overall decrease in Root Mean Square (RMS) roughness (Supporting Figure 37), while absorption measurements show only a slight reduction in PbI_2_-related absorbance after ligand treatment (Supporting Figure 38), indicating that the bulk PbI_2_ is largely preserved rather than significantly removed. Correspondingly, significant increases in surface CPD values indicated effective surface passivation by bidentate ligands (Figure 3d). Though showing smaller overall amplitude variations compared to both control and monodentate-treated films, MeX-treated films exhibited numerous localized high-CPD regions. Such residual heterogeneities, likely arising from localized grain-boundary traps or variations in interfacial dipole strength, may contribute to instability under harsh external stress.^56,57^ Conversely, the thiophene-based bidentate ligand MeXT yielded uniform dielectric coverage and spatially consistent CPD distributions, effectively neutralizing interfacial traps and establishing a coherent dipole across the entire surface (Figure 3d and Supporting Figure 39). Azimuthal intensity profiles derived from angle-dependent GIWAXS provide complementary structural insight into spatial uniformity (Supporting Figures 40, 41). Monodentate ligand treatment generated strong, highly anisotropic 2D perovskite diffraction signals oriented predominantly out-of-plane. By contrast, bidentate ligands yielded comparatively weaker yet more evenly distributed diffraction features across azimuthal angles, indicating isotropic and effective passivation in multiple crystallographic orientations.^58^ Together, bidentate ligand treatments point to a redistribution of PbI_2_ species at the surface rather than an etching-dominated mechanism.

A spatially resolved SPV map of pristine FAPbI_3_ reveals pronounced lateral inhomogeneity, reflecting spatially nonuniform surface electrostatics (Figure 3c). Monodentate ligand treatments fail to homogenize this response and instead introduce pronounced edge-localized features, either due to incomplete passivation of residual PbI_2_ or a less stable surface dipole formation, consistent with CPD maps (Figure 3b). In contrast, bidentate ligands yield SPV responses that are uniform across the film. Additionally, TRSPV measurements show that bare FAPbI_3_ exhibits an initial positive SPV, indicating hole migration to the surface (Figure 3e). After monodentate ligand treatment, this response surprisingly inverts to a pronounced negative SPV, indicating electron accumulation, which likely arises from ineffective passivation or even aggravation of residual PbI_2_-related defects. In sharp contrast, bidentate ligands fully restore and enhance the positive SPV response, again indicating preferential hole accumulation and efficient suppression of electron-trapped states. Notably, MeXT yields a positive SPV amplitude comparable to that of well-passivated PbI_2_ surfaces (Figure 2f), implying the formation of a spatially uniform, hole-favorable interfacial dipole without introducing additional trap states.

FLIM was used to probe recombination pathways associated with long-wavelength emission (>550 nm) (Supporting Figure 42). The longest lifetime composition distributions shift progressively toward longer lifetimes from monodentate to bidentate ligands, with MeXT exhibiting the most pronounced long-lived carrier population. Time-resolved photoluminescence (TRPL) measurements provide a complementary view of the overall recombination dynamics. The intensity-weighted lifetime τ_avg_ rises from 201 ns in pristine FAPbI_3_ to 329 ns with MeX and 437 ns with MeXT, indicating the suppression of the nonradiative pathway (Figure 3f and Supporting Table S4).

### Selective PbI_2_ Passivation by Bidentate Ligands Enables High-Efficiency and Stable Perovskite Photovoltaics

TRSPV measurements performed on perovskite/ligand/HTL stacks (Figure 4a) reveal trends consistent with PbI_2_/ligand (Figure 2f) and perovskite/ligand stacks (Figure 3d). Bidentate ligand-passivated devices exhibit a stronger and faster positive SPV response compared with monodentate-treated and control stacks, indicating more efficient hole extraction and reduced interfacial recombination at the perovskite/HTL interface. Due to the superior PbI_2_ selective passivation and inherent p-type nature of the bidentate ligands, we integrated them into n-i-p perovskite photovoltaic devices (Figure 4b). Statistical analyses confirmed a significant performance enhancement in devices passivated by bidentate ligands compared to monodentate-passivated and nonpassivated controls (Figure 4c). These improvements primarily originated from reduced defect densities and more favorable hole extraction at interfaces, resulting in pronounced increases in both open-circuit voltage (*V*_oc_) and fill factor (FF) (Figure 4d and Supporting Figure 43). The champion device passivated with MeXT exhibited a PCE of 26.19%, alongside a *V*_oc_ of 1.198 V, an FF of 83.2%, and a short-circuit current density (*J*_sc_) of 26.28 mA cm^–2^, which agrees with our TRSPV analysis (Supporting Figure 44). Additionally, the MeXT-passivated device maintained a stabilized PCE of 25.65% after 10 min of maximum power point (MPP) tracking (Supporting Figure 45). External quantum efficiency (EQE) measurements yielded an integrated current density of 25.6 mA cm^–2^, closely matching the *J–V* results (Supporting Figure 46).

**Figure 4:**
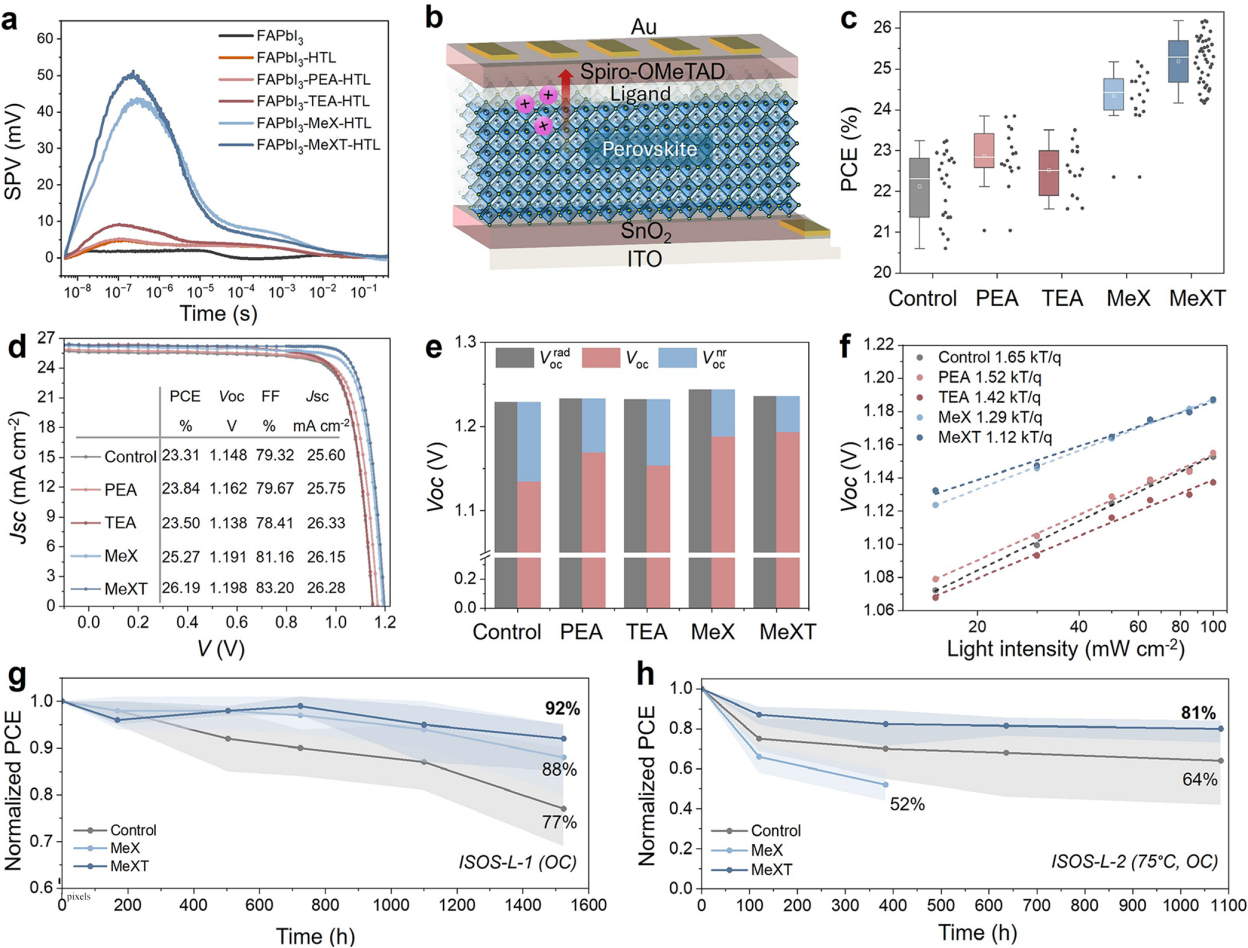
Device performance and nonradiative loss analysis. (a) TRSPV transients of ligand-passivated FAPbI_3_ films incorporated with hole transport layer. (b) Schematic of the n-i-p device architecture illustrating enhanced hole extraction at the perovskite/HTL interface, consistent with the increased SPV response in (a). (c) PCE statistical distribution. All boxes display the mean value, with 1.5× outlier range whiskers. (d) *J*–*V* curves of the champion devices with different ligand passivation. (e) Nonradiative *V*oc components from EQE plots. (f) *V*oc vs light intensity plots of devices with different ligand passivation. (g, h) Stability of PTAA-based devices under ISOS-L1 (OC, light) (g) and ISOS-L2 (75 °C, OC) (h). Initial PCEs (control/MeX/MeXT) are 18.97/21.08/21.75% in (g) and 16.91/21.18/20.50% in (h), respectively.

Further analysis of nonradiative voltage loss (*V*_nr oc_), calculated from EQE spectra, highlighted substantial reductions for bidentate-passivated devices, with *V*_nr oc_ decreasing from 0.094 V (control) to 0.055 V (MeX) and 0.042 V (MeXT) (Figure 4e). This improvement underscores the highly effective defect passivation and suppression of energetic disorder by bidentate ligands. Corresponding ideality factors, obtained from light intensity-dependent *V*_oc_ measurements, corroborate these trends (Figure 4f). Control and monodentate-treated devices exhibited relatively high ideality factors (1.65 to 1.42 kT/q), indicative of prevalent Shockley-Read-Hall (SRH) recombination. For bidentate ligand passivation, MeX and MeXT significantly lowered the ideality factors of devices to 1.29 and 1.12 kT/q, respectively, approaching values consistent with predominantly radiative recombination.^59^

The effective PbI_2_ passivation also markedly improved operational device stability. Under the ISOS-L-1 (OC) protocol, devices passivated with MeX and MeXT retained 88% and 92% of their initial efficiencies after 1500 h, respectively (Figure 4g). Furthermore, the MeXT-passivated device maintained exceptional stability under continuous maximum power point tracking (ISOS-L-1 (MPP)) (Supporting Figure 47). Thermal stability under illumination, monitored under the ISOS-L-2 protocol (75 °C, OC), demonstrated that the MeXT-passivated device maintained over 80% of its initial efficiency after 1000 h of aging. Interestingly, MeX-passivated devices exhibited less stable performance under prolonged thermal and photonic stress, consistent with spatial inhomogeneities in surface contact potential revealed earlier by KPFM analysis (Figure 4h).

## Conclusions

In this work, we introduced a rational ligand engineering approach centered around structurally tailored bidentate ligands designed for targeted PbI_2_ passivation in metal halide perovskite solar cells. Our systematic comparative studies showed that bidentate ligands selectively and robustly coordinate PbI_2_ residues, suppressing their aggregation and stabilizing dispersed Pb–I motifs without disturbing the underlying 3D perovskite structure. Unlike monodentate analogues, bidentate ligands yield spatially uniform surface passivation and homogeneous electronic environments. This results in significantly reduced interfacial defect densities, substantially lowered nonradiative recombination losses, and strongly enhanced hole extraction, thus enhancing device open-circuit voltage, fill factor, and overall photovoltaic performance. Devices incorporating thiophene-terminated bidentate ligand passivation achieved a high PCE of 26.19%, demonstrating exceptional operational stability, with negligible efficiency degradation after 1000 h of continuous illumination and thermal stress. Beyond performance enhancement, this work establishes a generalizable framework for regulating spatial energetics through selective interfacial chemistry, offering a pathway to address photostability challenges associated with residual PbI_2_ in perovskite optoelectronic devices.

## Methods

### Materials

All reagents were used as received. SnO_2_ colloid (Alfa Aesar); PbI_2_, 99.99% trace-metal grade (Fisher Scientific); Formamidinium iodide (FAI), methylammonium chloride (MACl), phenethylammonium iodide (PEAI), and thiopheneethylammonium iodide (TEAI) (GreatCell Solar); and PTAA (Ossila); KCl, methylenediamine dihydrochloride (MDACl_2_), Spiro-OMeTAD (SHT-263S), bis­(trifluoromethane)­sulfonimide lithium salt (Li-TFSI), 4-*tert*-butylpyridine (tBP), tris­(pentafluorophenyl)­borane (TPFB), dimethylformamide (DMF), dimethyl sulfoxide (DMSO), isopropyl alcohol (IPA), chlorobenzene (CB), acetonitrile (ACN) and diethyl ether (DEE) were sourced from Sigma-Aldrich. Gold pellets were obtained from Kurt J. Lesker. The synthesized route for bidentate ligands was reported in the Supporting Information.

### PbI_2_ Film Study

0.5 M PbI_2_ in DMF was prepared and stirred at 70 °C for 1 h before use. The PbI_2_ solution was then spin-coated on ITO or glass substrates at 1500 rpm for 30 s, and annealed at 70 °C for 1 min. After cooling down, the film was soaked by 0.5 mg mL^–1^ ligand in IPA/CB (1:9 v/v) for 1 min, followed by spin-coating for another 30 s.

### Film Characterization

Solution-state UV–vis spectra were recorded on an Agilent Cary 5000 spectrophotometer using the IPA/CB (1:9 v/v) solvent mixture as the reference blank.

Ultraviolet photoelectron spectra (UPS) were recorded under ultrahigh vacuum (base pressure <1 × 10^–9^ mbar) with a −7 V bias applied to the sample, a hydrogen Lyman-α source (E-LUX-121, *hν* = 10.2 eV) for excitation, and a PHOIBOS 100 hemispherical analyzer with a 2D CMOS detector. X-ray photoelectron spectra (XPS) were acquired on the same ultrahigh vacuum system using a dual-anode Mg Kα source (*hν* = 1253.64 eV) and the same PHOIBOS 100/150 hemispherical analyzer. Satellite features occasionally visible in the XPS traces arise from shakeup/shake-off events and multiple splitting inherent to the photoemission process. Elemental atomic fractions were obtained from background-subtracted peak areas (A_*i*_) by

$$\frac{\mathrm{Pb}}{I}=\frac{\sum \frac{A_{\mathrm{Pb}}}{{\mathrm{RSF}}_{\mathrm{Pb}}}}{\sum \frac{A_{I}}{{\mathrm{RSF}}_{I}}}$$

where RSF_*i*_ are the instrument-specific relative sensitivity factors.

Optical and photoluminescence (PL) micrographs were obtained with a customized Olympus BX53 microscope illuminated by an X-Cite 120 lamp (012–63000), and the corresponding PL spectra were recorded using a SpectraPro HRS-300 spectrometer.

Atomic Force Microscopy (AFM) was performed on Park Systems NX-10 in noncontact mode using Ti/Ir-coated silicon cantilevers (ASYELEC.01-R2) mounted in a compatible chip carrier. Kelvin Probe Force Microscopy (KPFM) was conducted with the same tip, operated at a lift height of 15 nm. A drive voltage of 1.0 V was applied to the conductive tip. Images were acquired over scan sizes of 10 μm at a resolution of 256 × 256 pixels, with a scan rate of 1.0 Hz. AFM and KPFM images were processed using Gwyddion for flattening and statistical analysis.

X-ray diffraction (XRD) patterns were recorded on a Rigaku SmartLab with a 9 kW Cu Kα rotating-anode source (λ = 1.54178 Å), five-axis in-plane goniometer, and HyPix-3000 2D detector. Scanning electron microscope (SEM) images were captured using a Hitachi S-4800 SEM operating at 5 kV, with a secondary electron and backscattered electrons mixed detector. Grazing-Incidence Wide-Angle X-ray Scattering (GIWAXS) was carried out at ALS beamline 7.3.3 using a 10 keV beam and a 0.1° incident angle. FTIR spectra were recorded with a Thermo Nicolet Nexus spectrometer.

Transient surface photovoltage (TRSPV) was recorded using a setup built in-house on encapsulated samples. The samples were excited from the top surface by a tunable pumped pulse laser (Nd:YAG Laser, EKSPLA, NT230–50-SH/SF-SCU-2H) with a pulse time of 3–5 ns, a frequency of 2 Hz, and a spot size of ∼0.2 cm^2^ The excitation energy was varied from 1.4 eV (885 nm) to 2.4 eV (516 nm) in 0.2 eV increments, at an induced charge carrier density comparable to 1 sun illumination. For each excitation energy, 30 curves were measured and averaged via an oscilloscope card (Gage, CSE 1622–4GS, 200 MS s^–1^) using a software for logarithmic read-out developed in-house.

SPV mapping was recorded on a custom-made setup, using laser pulse lengths of 200 μs to ensure that a steady-state SPV was reached during each pixel dwell. For perovskite films, a 1.6 eV (780 nm) LED laser was used, while PbI_2_ films were mapped using a 2.8 eV (447 nm) laser. The samples were excited from the top surface with a laser intensity of 0.23 mW and a spot size of 0.99 mm^2^. For every pixel of each sample, 500 transients were recorded and averaged. Unlike the trSPV setup, SPV mapping cannot distinguish positive from negative amplitudes. All amplitudes were normalized to show homogeneity of the SPV distribution within each sample instead of implying comparability of the values between samples.

Fluorescence lifetime imaging microscopy (FLIM) was performed using a Nikon TE2000 confocal microscope equipped with a 60×/1.2 NA water-immersion objective and an Alba FastFLIM system (FFS), as previously described.^60,61^ Samples were excited with a 440 nm pulsed laser operating at a modulation frequency of 1 MHz. Fluorescence emission above 550 nm was collected through a 550 nm long-pass filter and detected using MPD avalanche photodiode (APD) detectors. Acquired FLIM images were subsequently analyzed in VistaVision software (VVS) using a three-component exponential decay fitting model at each pixel to characterize the fluorescence lifetime distribution of the synthesized materials.

TRPL was measured using a home-built confocal PL microscope. A 480 nm, 500 nW pulsed laser operating at 250 kHz was used as the excitation light source (Pharos operated with TOPAS-Twins optical parametric amplifier, Light Conversion). A 40x, NA = 0.6 objective was used to focus the excitation laser and collect the emission from the perovskite thin film. The emission was then measured by a single-photon avalanche diode operating with a single-photon counting module (PicoQuant) at a resolution of 64 ps. Decay curves were fitted with a triexponential model

$$I\left(t\right)=\sum_{n}\left(A_{n}\cdot \exp\left(-\frac{t}{\tau_{n}}\right)\right)+c$$

### Device Fabrication

Inside a N_2_ glovebox, we prepared the perovskite ink by dissolving PbI_2_ (726 mg, 5% excess), FAI (258 mg), MACl (34.4 mg), and MDACl_2_ (6.8 mg) in the mixed solvent containing 888 μL DMF and 112 μL DMSO and stirring for 2 h. Patterned ITO substrates were sequentially sonicated for 15 min each in soap water, deionized water (DIW), acetone, and IPA, and UV-ozone treated for 20 min before use. The substrate was then spin-coated with SnO_2_ colloid (SnO_2_/DIW/IPA = 1:3:2 v/v, 3000 rpm 30 s) and annealed at 150 °C for 30 min. 50 mM KCl (3000 rpm 30 s) was then spin-coated on SnO_2_ for passivation. The perovskite layer was deposited by spinning the ink at 1000 rpm for 10 s, then 5000 rpm for 15 s while dropping diethyl ether at 15 s, followed by air annealing at 150 °C for 10 min (40% RH). Back in the glovebox, films were passivated with 0.5 mg mL^–1^ ligand in IPA/CB (1:9 *v*/*v*) at 4000 rpm for 30 s. For the hole-transport layer, either Spiro-OMeTAD (51.5 mg in 600 μL CB, 20.3 μL *t*BP and11.7 μL Li-TFSI in ACN, 520 mg mL^–1^) or PTAA (24 mg in 600 μL CB and 26.6 μL TPFB in ACN, 100 mg mL^–1^) was spin-coated at 3000 rpm for 30 s, with PTAA layers additionally annealed at 80 °C for 5 min. Finally, 90 nm of Au was thermally evaporated at 0.3 Å s^–1^ below 2 × 10^–6^ Torr to complete the devices. The active area for the device is around 0.5 cm^2^. The active area is determined by optical microscopy. The devices were aged 3 days in a dry cabinet (<5 RH%) before measuring.

### Device Characterization

*J*–*V* characteristics and device efficiency were measured in a N_2_ glovebox under AM 1.5 G, 1-sun illumination provided by an Enlitech SS-F5–3A solar simulator, which was calibrated using a certified silicon reference cell (NREL) prior to measurements. The voltage was scanned from 1.22 V to −0.10 V and then back to 1.22 V (reverse–forward scan). A nonuniform step size was applied, with 10 mV steps from 1.22 to 0.80 V and 20 mV steps from 0.80 to −0.10 V using the advanced measurement function. The delay time was set to 10 ms per step, and no preconditioning was applied. External quantum efficiency (EQE) was recorded in air at zero bias using a custom system with a preamplifier, lock-in amplifier (161 Hz chopper), and source calibration via a Si photodiode (Thorlabs 818-UV-L).

## Supplementary Material





## Data Availability

Crystallographic data for the (MeXT)­PbBr_4_ with structural disorder reported in this Article have been deposited at the Cambridge Crystallographic Data Centre (CCDC), under deposition number CCDC 2548865. Copies of the data can be obtained free of charge via https://www.ccdc.cam.ac.uk/structures. The other data that support the findings of this study, including synthesis route, computational methods, and additional experimental details, are available within the Supporting Information.

## Notes

Synthesis and characterization of MeXT and MeXTBoc; ^1^H NMR spectra; DFT calculations, adsorption-energy analysis, crystal-structure prediction, and machine-learning interatomic potential simulations; single-crystal structure of (MeXT)­PbBr_4_; UV–vis absorption, photoluminescence, spatially resolved PL mapping, SEM, AFM, XPS, UPS, FTIR, XRD, GIWAXS, and KPFM characterization of ligand-treated PbI_2_ and FAPbI_3_ films; adsorption configurations and surface electronic structure analysis; photovoltaic device fabrication and characterization; *J*–*V* characteristics, EQE spectra, stability measurements; and statistical device performance; supporting figures, tables, and references (PDF)
Bidentate ligands yielded uniform fluorescence across the entire film, suggesting more homogeneous interaction with the PbI_2_ surface (ZIP)
