# Supplementary material for: Selective Bidentate Coordination Reconstructs Residual PbI2 to Homogenize Interfacial Energetics in Perovskite Solar Cells
Source: J Am Chem Soc. 2026 Jul 8;148(28):29921–31. doi: 10.1021/jacs.6c05316 (PMC13397558; doi:10.1021/jacs.6c05316)
Supplement: Supplementary file 1 [file ja6c05316_si_001.pdf]

Supporting Information

Selective bidentate coordination reconstructs residual PbI_2 to homogenize interfacial energetics in perovskite solar cells

Yuanhao Tang[1,2]†, Chenjian Lin[1,2]†, Zhichen Nian[1,3]†, Thomas W. Gries[4], Jeong Hui Kim[1,2], Kevin R. Pedersen[5], Yu-Ting Yang[1,2], Siddha Hill[4], Vignesh Sathyaseelan[1], Yunfei Wang[6], Hanjun Yang[1,7], Han Zhao[1], Wenzhan Xu[2], Zheng-Fei Liu[2], Xiyu Luo[2], Yanyan Li[2], Chongli Yuan[1], Libai Huang[7], Chenhui Zhu[6], Kenneth R. Graham[5], Artem Musiienko[4*], Brett M. Savoie[1,3*], Letian Dou[1,2*]

[1]Davidson School of Chemical Engineering, Purdue University, West Lafayette, IN 47907, USA

[2]Department of Chemistry, Emory University; Atlanta, GA 30322, USA

[3]Chemical and Biomolecular Engineering, University of Notre Dame; South Bend, IN 46556, USA

[4]Robotized Optoelectronic Material and Photovoltaic Engineering, Helmholtz-Zentrum Berlin für Materialien und Energie (HZB), Berlin 12489, Germany

[5]Department of Chemistry, University of Kentucky; Lexington, KY 40506, USA

[6]Advanced Light Source, Lawrence Berkeley National Laboratory, Berkeley, CA 94720, USA

[7]Department of Chemistry, Purdue University, West Lafayette, IN 47907, USA

*Corresponding authors. Email: L.D. letian.dou@emory.edu; B.M.S. bsavoie2@nd.edu; A.M. artem.musiienko@helmholtz-berlin.de

†These authors contributed equally to this work.

The PDF file includes:

Synthesis

Computational Method

Supplementary Figs. 1 to 47

Supplementary Tables 1 to 4

Other Supplementary Information for this manuscript includes the following:

Supplementary Movies 1 to 4

Synthesis

All the chemicals were purchased from the chemical suppliers (Sigma Aldrich, Fisher Scientific, Ambeed) and used without further purification unless otherwise specified. The silica gel (200-300 mesh) for column chromatography was purchased from Natland International Corporation. All the ^{1}H-NMR spectra were collected on the Bruker Avance DRX-500-1 system at the Purdue Interdepartmental NMR Facility (PINMRF). Deuterated solvents for NMR ($CDCl_3$, DMSO-d_6) were purchased from Cambridge Isotope Laboratories Inc. Synthesis of **(MeX)I** was included in a manuscript under review[1]. Precursors of the **MeXTBoc** reaction were synthesized according to the previously reported procedures[2,3] with modifications.

Synthesis of **MeXTBoc**. *tert*-butyl (2-(5-(trimethylstannyl)thiophen-2-yl)ethyl)carbamate (2.30 g, 5.90 mmol), 4,5-dibromo-2,7,9,9-tetramethyl-9H-xanthene (500 mg, 1.26 mmol), Tris(dibenzylideneacetone)dipalladium(0) (46 mg, 0.050 mmol), Tri(o-tolyl)phosphine (61 mg, 0.20 mmol) were added into a 500-mL 3-neck round-bottom flask. The system was purged with argon and injected with toluene (200 mL). The system was then heated to 100 ^{o}C for 18 h. After condensing the organic solvents, the crude product was diluted with dichloromethane and washed with deionized water. The organic layer was dried with sodium sulfate and condensed to yield the crude product. The crude product was purified by chromatography with dichloromethane and ethyl acetate (15:1 to 10:1 ratio). The resulting light-yellow oil was dried under vacuum and yielded 360 mg (41%). ^{1}H NMR (500 MHz, $CDCl_3$) δ 7.16 (d, J = 2.1 Hz, 2H), 7.12 (d, J = 2.1 Hz, 2H), 6.92 (d, J = 3.5 Hz, 2H), 6.65 (d, J = 3.5 Hz, 2H), 3.48 – 3.36 (m, 4H), 2.99 (t, J = 6.9 Hz, 4H), 2.34 (s, 6H), 1.65 (s, 6H), 1.45 (s, 18H).

Synthesis of MeXTI. MeXTBoc, (182 mg, 0.264 mmol), hydroiodic acid (57 wt. % in H_2O, 173 mg, 0.771 mmol), and hypophosphorous acid (14.9 mg, 0.226 mmol) were added to a 100-mL round-bottom flask. The system was purged with argon and injected with ethanol (10 mL) and chloroform (2.0 mL). The system was then heated to 55 ^{o}C for 26 h. After condensing the solvents, 50 mL of diethyl ether was added to precipitate out the crude product. The crude product was obtained by filtration and purified by sonicating in diethyl ether (50 mL x3). The resulting white solid product was dried under vacuum and yielded 184 mg (94%). ^{1}H NMR (500 MHz, DMSO) δ 7.85 – 7.74 (m, 6H), 7.37 (d, J = 2.1 Hz, 2H), 7.12 (d, J = 2.1 Hz, 2H), 6.98 (d, J = 3.5 Hz, 2H), 6.82 (d, J = 3.5 Hz, 2H), 3.07 (d, J = 5.5 Hz, 8H), 2.32 (s, 6H), 1.62 (s, 6H).

Computational Method

Density functional theory (DFT) calculations

Molecular

DFT calculation was performed using Gaussian16 (Revision B-0.1).[4] Calculations were carried out using the long-range corrected hybrid functional ωB97X-D[5], which accounts for dispersion interactions, in conjunction with the 6-311G(d,p) basis set.[6] To visualize charge distribution, the Molecular Electrostatic Potential (MESP) surfaces and potentials were visualized using VMD (version 1.9.3).[7]

Surface and adsorption

All DFT calculations were performed using the Vienna *ab initio* simulation package (VASP)[8-10] with the projector-augmented wave (PAW) method[11]. The Perdew–Burke–Ernzerhof (PBE)[12] exchange–correlation functional within the generalized gradient approximation (GGA) was employed. Valence electrons were represented with a plane-wave basis set with an energy cut-off of 500 eV. Structural optimizations were converged until the residual force on each atom was below 0.02 eV/Å and the total energy change between ionic steps was smaller than 10^{-6} eV. Long-range dispersion interactions were included using the DFT-D3[13] van der Waals correction. A vacuum spacing of 20 Å was applied along the surface normally to avoid spurious interactions between periodic images of the slab. Brillouin-zone sampling was performed using a Γ-centered 3 × 3 × 1 k-point mesh for the adsorption systems, as well as for the isolated slabs and ligands.

Slab models of PbI_2 and FA-terminated $FAPbI_3$ were constructed using two atomic layers. During structural relaxation, the simulation cell vectors were kept fixed, and the bottom layer of the slab was held fixed to mimic the underlying bulk constraint while allowing the surface layer and the adsorbate to fully relax.

Adsorption energy calculation

The adsorption energy was defined as:

$$E_{ads} = E_{total} - E_{slab} - E_{ligand}$$

where E_{ads} is the adsorption energy, E_{total} is the total energy of the fully relaxed adsorption system, and E_{slab} and E_{ligand} are the energies of the isolated substrate and ligand, respectively. More negative E_{ads} values indicate stronger surface adsorption. To maintain a consistent reference, the slab and ligand geometries were extracted from the relaxed adsorbed configuration and then individually re-optimized using the same computational settings as the adsorption system. The relaxed structures were shown in **Supplementary Figs. 5,6**.

To identify representative low-energy adsorption conformations prior to full DFT relaxation, a conformational sampling procedure was used to generate candidate ligand–surface configurations across different surface binding sites. Here, the experimentally resolved crystal structures of PbI_2 and $FAPbI_3$[14,15] were adopted to construct the PbI_2 surface and the FA-terminated $FAPbI_3$ surface. For each substrate, three adsorption sites were explored using standard surface-science terminology. For PbI_2, initial ligand placement was performed at an atop site directly above a

terminal surface iodide atom, a bridge site located at the midpoint between two neighboring surface iodide atoms, and a hollow site corresponding to the center of the triangular motif defined by the three nearest surface iodide atoms. On FA-terminated $FAPbI_3$, ligand placement was similarly explored at an atop site above a surface iodide, a bridge site between two surface iodides, and a fourfold hollow/vacancy-like site corresponding to the region above a four-iodide opening on the surface.

For each binding site, the ligand was initially positioned 3.0–4.2 Å above the surface. This range was chosen based on the sum of the van der Waals radii of the ligand anchoring N atom and the surface adsorption I atom, with an additional ±0.5 Å perturbation. Candidate geometries were then generated by randomized orientational sampling. Specifically, the ligand was rotated about the surface normal (z-axis) by a randomly chosen angle between 0° and 360° and tilted relative to the surface using a set of predefined tilt angles. Each candidate geometry was screened to remove unphysical structures, rejecting structures with short interatomic contacts indicative of steric clashes. A total of 100 candidate adsorption structures were generated and evaluated using single-point semiempirical xTB calculations[16] performed with CP2K[17] using an electronic convergence criterion of 10^{-5} eV. The three lowest-energy conformers from this screening step were then selected as starting geometries for subsequent DFT structural relaxations.

Predicted crystal structure for $MeXTPbI_4$ and $MeXTPbBr_4$

Because experimentally determined crystal structures of $(MeXT)PbI_4$ and $(MeXT)PbBr_4$ are not available, initial structural models were constructed from the reported crystal structures of the corresponding $(MeX)PbI_4$ and $(MeX)PbBr_4$ compounds, following the approach used in our previous work.[1]

The parent (MeX)-based structures were first preprocessed at the xTB level in two steps. In the first step, the experimentally resolved crystal structures were refined to remove atomic overlaps. In the second step, solvent molecules were removed from the structures. After each step, geometry optimization was performed with CP2K[17] at the GFN1-xTB[16] level of theory. The convergence criteria were set to 0.02 eV/Å for the maximum force and 1×10^{-5} eV for the total energy.

The refined $(MeX)PbI_4$ and $(MeX)PbBr_4$ structures were then used as templates to generate the corresponding $(MeXT)PbI_4$ and $(MeXT)PbBr_4$ models. This was done by modifying the MeX^{2+} ligands to include the ligand-specific structural features of $MeXT^{2+}$ while preserving the overall framework of the crystal structure.

To evaluate the structural stability of the predicted MeXT-based 2D perovskites, full geometry optimization was carried out again at the GFN1-xTB level using CP2K. These calculations were used to determine whether the predicted structures remained close to the original MeX-based bidentate perovskite templates or underwent substantial structural distortion upon relaxation. The same convergence criteria were applied in these calculations. The optimized structures are summarized in **Supplementary Figs. 7**.

Machine-learning interatomic potential calculations

To probe how mono- and bidentate ligands interact with (and potentially react on) PbI_2 and FAPbI3 surfaces, three machine learning interatomic potentials (MLIPs) were used: MACE-MPA-0[18], Orb-v3[19], and UMA-S-1.1[20], coupled with the Atomic Simulation Environment (ASE)[21] as the simulation engine. These pre-trained models share several architectural features, most notably the treatment of charge as a global input feature without the resolution of explicit atomic partial charges or the use of charge-equilibration schemes. MACE utilizes a rotationally equivariant atomic cluster expansion with message passing, while Orb-v3 is built upon a sparse graph neural network with roto-equivariant embeddings and an additional Ziegler–Biersack–Littmark (ZBL) potential[22] to account for short-range repulsive forces. UMA-S-1.1 employs a modified equivariant Smooth Energy Network (eSEN) architecture utilizing equivariant spherical convolutions. All three models utilize a 6 Å neighbor interaction cutoff. MACE-MPA0 was trained on the MPtrj and Alexandria datasets, while Orb-v3-conservative-inf-omat utilized a combined dataset of MPtrj, Alexandria, and Omat24, and UMA-S-1.1-omat was trained on Omat24[23-25].

PbI_2 surface chemistry

Using each MLIP, geometry optimizations of PbI_2 slab were performed both in vacuo and in a solvated environment (isopropanol and chlorobenzene in a 1:9 ratio) using the BFGS optimizer as implemented in ASE (**Supplementary Fig. 9a, b**). Subsequent finite-temperature molecular dynamics simulations were conducted using ASE. Initial atomic velocities were drawn from a Boltzmann distribution at 300K, followed by sampling within the NVT ensemble using a Langevin thermostat at 300K with a friction coefficient of 0.01 fs-1employing a 0.5 fs timesteps for a total of 300 ps (**Supplementary Fig. 9c**).

Among these three MLIPs tested, only the MACE model preserved the layered PbI_2 motif during 0 K geometry optimization (**Supplementary Fig. 9b**). However, none of the evaluated MLIPs produced stable finite-temperature dynamics for the PbI_2 slab under the MD conditions used here, and structural degradation was observed during the trajectories (**Supplementary Fig. 9c**).

Crystal seed in solution

Crystal seeds were constructed using two protocols. In the crystal-derived protocol, layered perovskite structures of $(PEA)_2PbI_4$ and $(TEA)_2PbI_4$ were obtained from experimental crystallographic data[26,27]. The structure of $(MeX)PbI_4$ was taken from our previous work[1], and $(MeXT)PbI_4$ was generated by templating from $(MeX)PbI_4$, following the same procedure described in[21]. All crystal structures were geometry-optimized using VASP with the same computational settings described in the adsorption energy calculation section. From each optimized layered structure, one $[PbI_6]^{4-}$ octahedral unit together with its nearest coordinating ligands was extracted to form the initial seed, and additional iodide ions were introduced when necessary to ensure overall charge neutrality (**Supplementary Fig. 10a**).

In the manually built protocol, a single octahedral core was constructed with all Pb–I bond lengths set to 3.1 Å, and ligand coordination motifs were imposed explicitly. For monodentate ligands, four ligands were placed to cap the octahedron, with two ligands oriented downward and the other two oriented upward along opposing diagonal directions. For bidentate ligands, two ligands were arranged to cap the top and bottom faces of the octahedron, respectively.

Seed-in-solution configurations were generated by first placing solvent molecules on a regular grid within a 40 × 40 × 40 $Å^3$ simulation cell. The solvent consisted of chlorobenzene (CB) and isopropanol (IPA) with a 9:1 volumetric ratio (CB: IPA) to match experimental conditions. The crystal seed was then inserted into the solvated box, and overlapping solvent molecules were removed; a solvent molecule was classified as colliding and deleted if any of its atoms were within 2 Å of the seed (**Supplementary Fig. 10b**).

For the crystal seed, only MACE-MPA-0 and UMA-S-1.1 potential were used due to the unsatisfactory result Orb-v3 shown in sample tests. All simulations were conducted under periodic boundary conditions (PBC). Each system was equilibrated in the NVT ensemble using a Langevin thermostat for 20 ps, with the total linear momentum removed every 1 ps, followed by a second NVT equilibration at 300 K using a Langevin thermostat with a relaxation time of 300 ps. The integration timestep of 1 fs was used throughout, and atomic configurations were saved every 1000 steps (1 ps).

In the MLIP MD trajectories, several unphysical artifacts were observed, indicating potential out-of-distribution behavior for the present systems. For example, in simulations initiated from the crystal-derived MeX seed, two iodide ions formed a persistent I–I "bond" with an interatomic distance of ~2.9 Å for >30 ps (**Supplementary Fig. 10c**). In addition, 0 K BFGS optimization of PbI_2 yielded a strongly distorted structure for some MLIPs. These observations suggest that current general-purpose MLIPs may not reliably describe the uncommon coordination environments explored here, and that further methodological advances are needed to accurately model these systems.

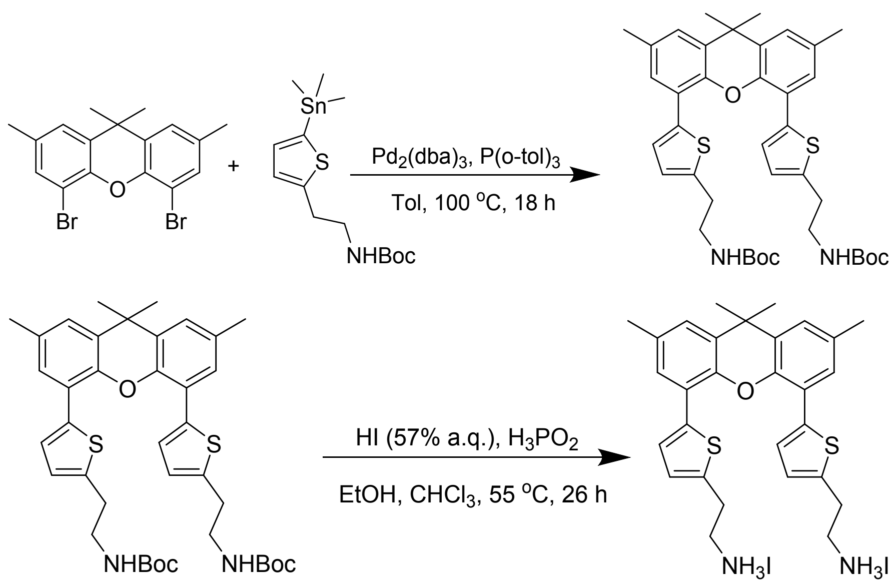

Supplementary Fig. 1 | Synthesis route for MeXT.

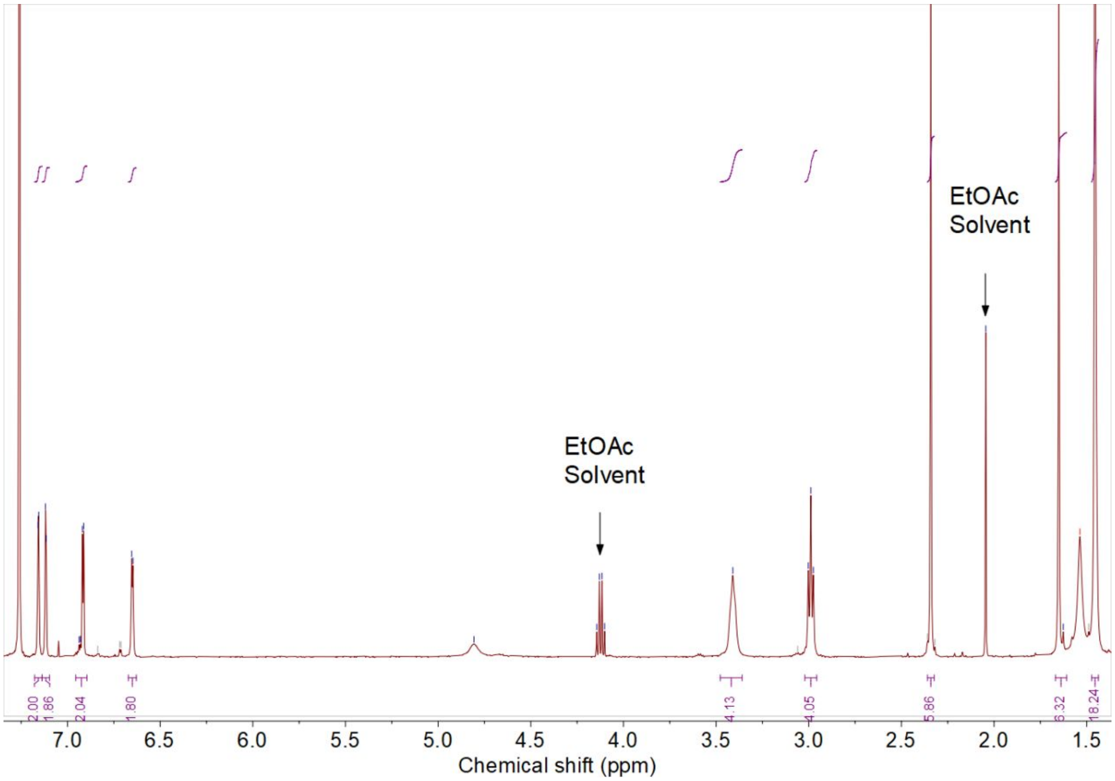

Supplementary Fig. 2 | 1H-NMR spectrum of MeXTBoc.

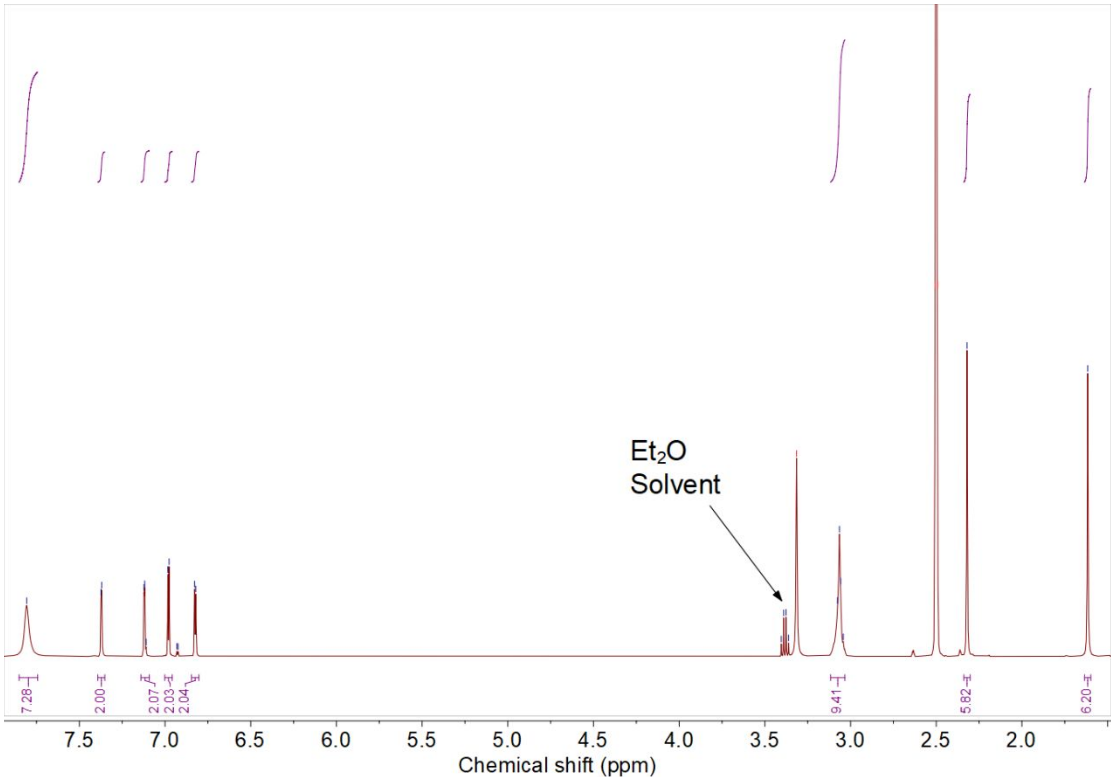

Supplementary Fig. 3 | 1H-NMR spectrum of MeXT.

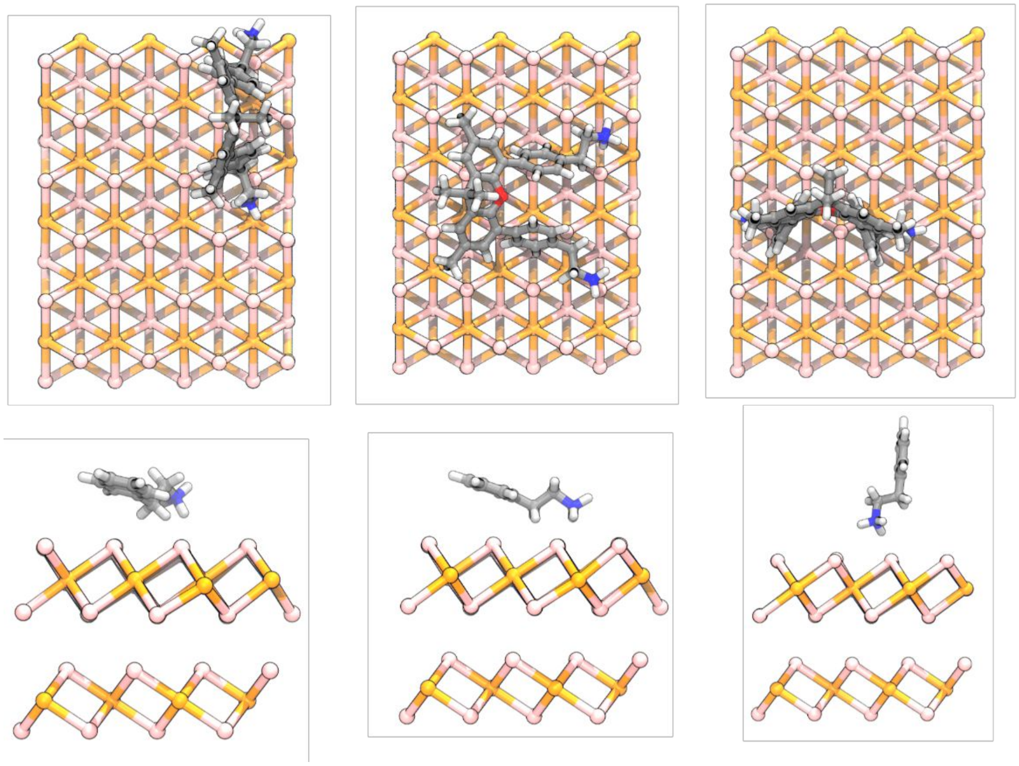

Supplementary Fig. 4 | Relaxed adsorption system for MeX (top) and PEA (bottom) on PbI_2.

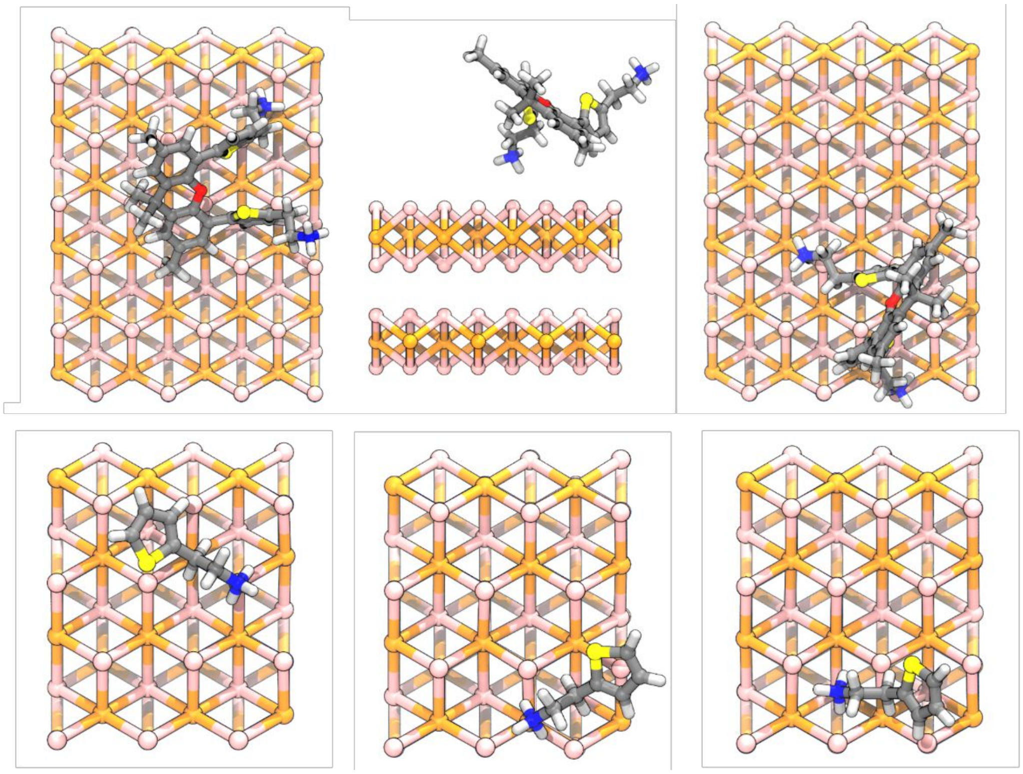

Supplementary Fig. 5 | Relaxed adsorption system for MeXT (top) and TEA (bottom) on PbI_2.

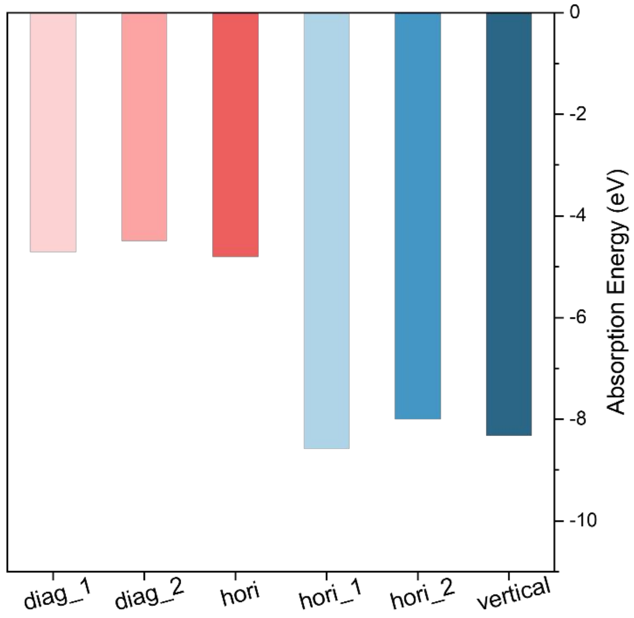

Supplementary Fig. 6 | Simulated adsorption energies of TEA and MeXT on PbI_2 surfaces.

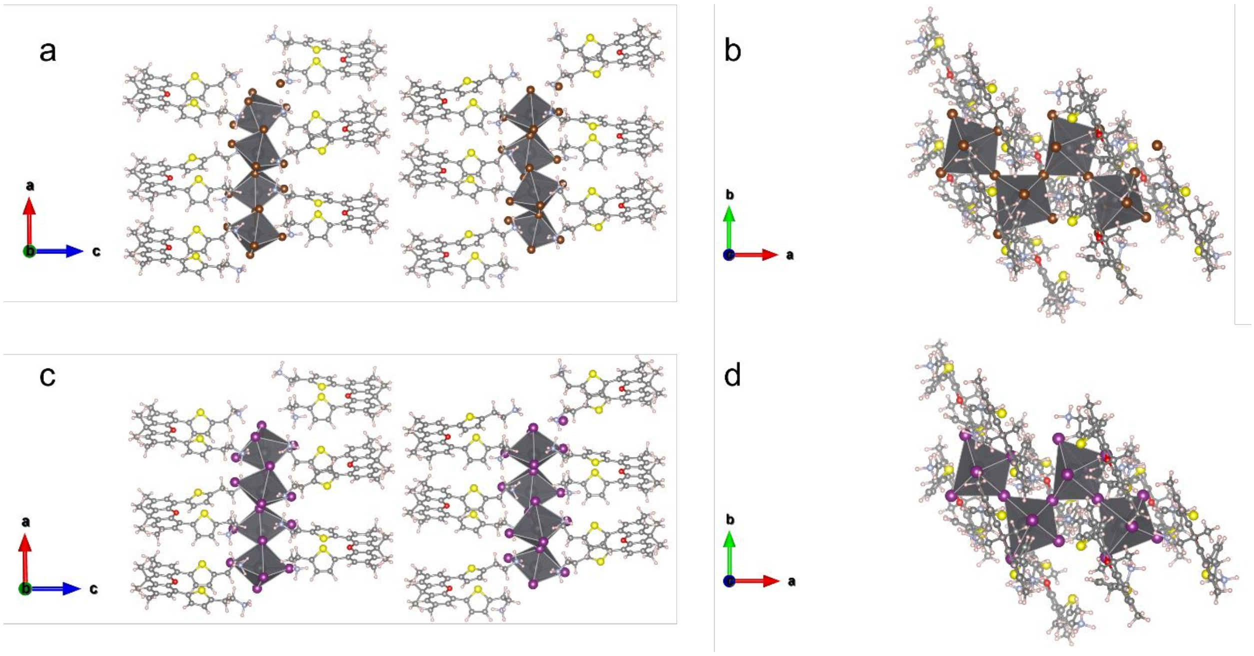

Supplementary Fig. 7 | Crystal structures of MeXT-based perovskites derived from DFT calculations. (a,b) Side and top views of $(MeXT)PbBr_4$. (c,d) Side and top views of $(MeXT)PbI_4$. Atoms: C, gray; H, white; N, light blue; S, yellow; O, red; Br, brown; I, purple; PbX_6 (X = I or Br), dark gray octahedra.

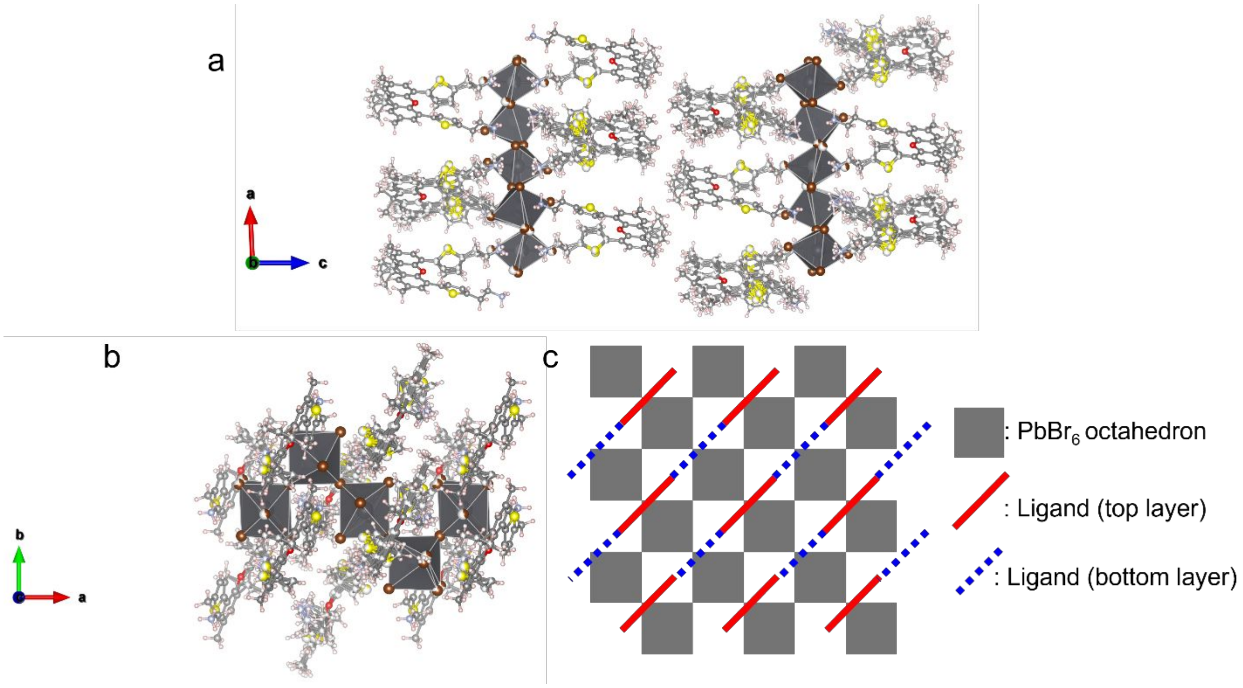

Supplementary Fig. 8 | Single-crystal structure of (MeXT)$PbBr_4$ with structure disorder. (a) Side view and (b) top view of the crystal structure, showing a layered arrangement of corner-sharing $PbBr_6$ octahedra separated by MeXT. Notably, some defect sites are present in the crystal structure; however, the bidentate anchoring motif is consistently observed, with both ammonium groups anchoring to the inorganic framework. (c) Schematic illustration of the double-point ligand-framework interaction. Atoms: C, gray; H, white; N, light blue; S, yellow; O, red; Br, brown; $PbBr_6$, dark gray octahedra.

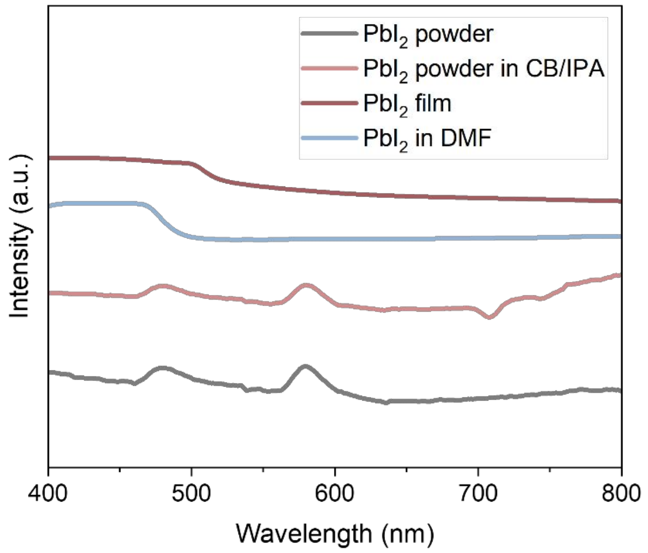

Supplementary Fig. 9 | UV–vis absorption spectra of PbI_2 in different physical states, including powder, powder dispersed in CB/IPA, thin film, and solution in DMF.

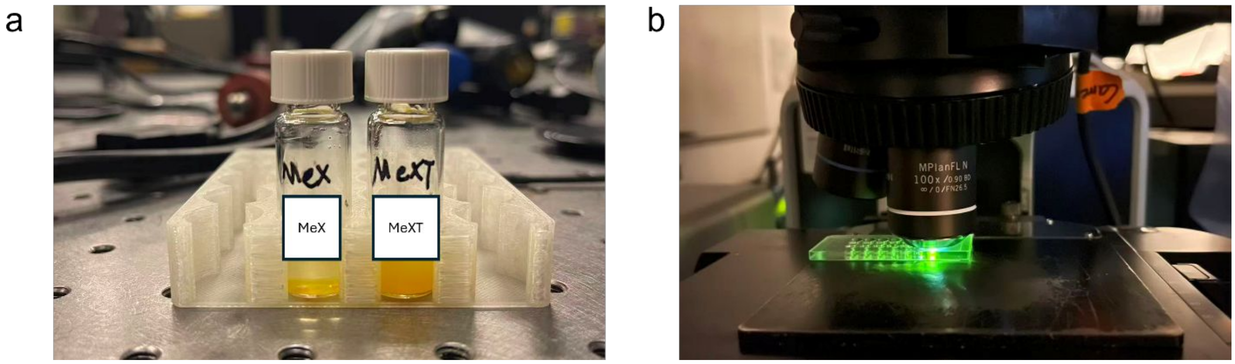

Supplementary Fig. 10 | (a) Photograph of MeX- and MeXT-incorporated PbI_2 solutions prepared in CB/IPA, exhibiting distinct coloration indicative of ligand–Pb complexation. (b) Representative experimental setup for liquid-phase PL spectroscopy under 375 nm laser excitation. PbI_2-MeXT solution shows strong green luminescence.

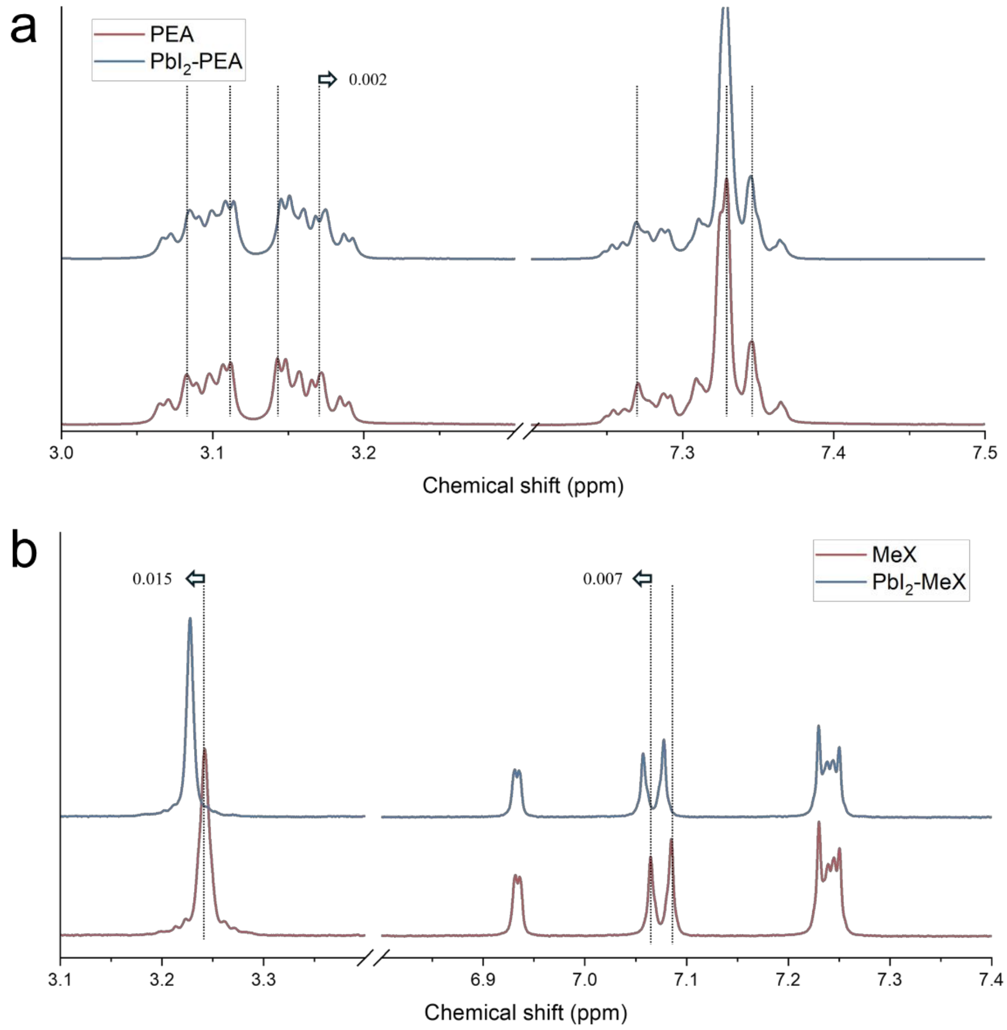

Supplementary Fig. 11 | 1H NMR of PEA (a) and MeX (b) incorporated with PbI_2 in deuterated isopropanol.

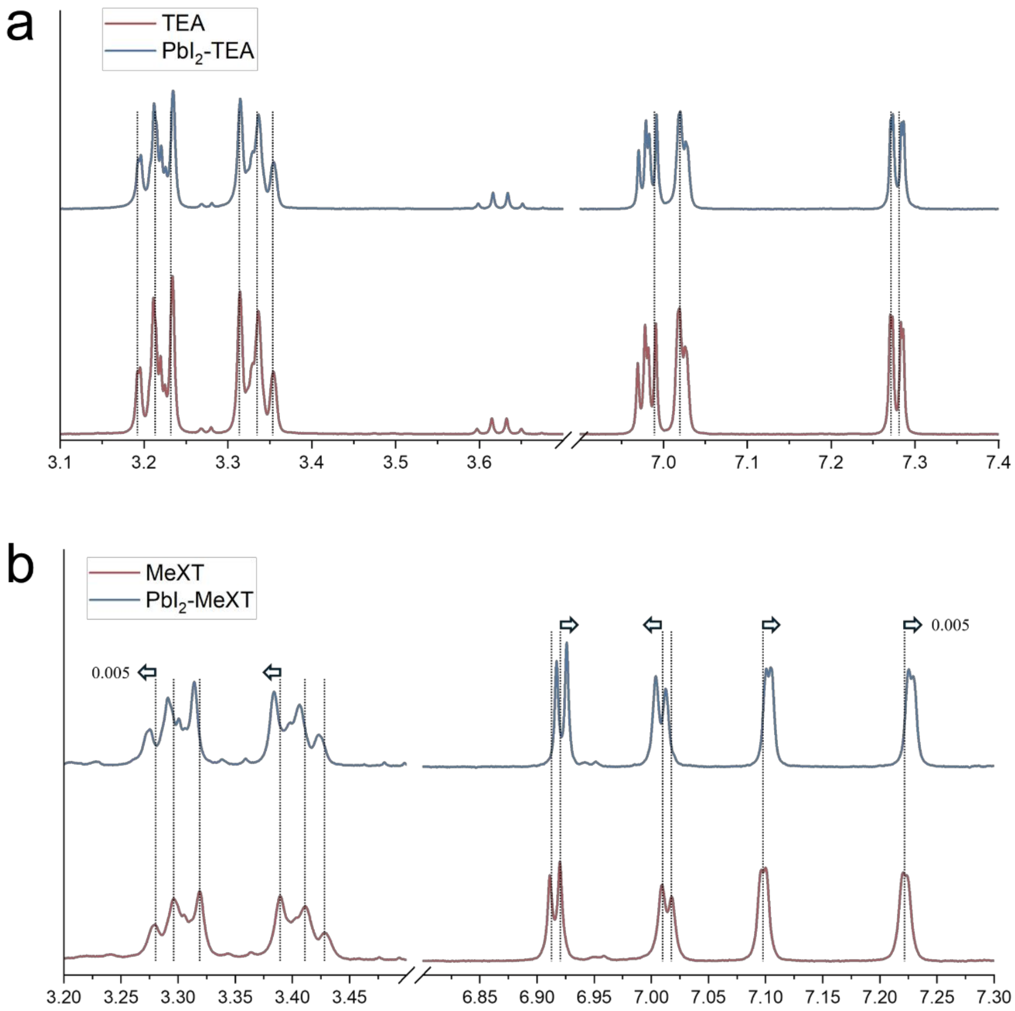

Supplementary Fig. 12 | 1H NMR of TEA (a) and MeXT (b) incorporated with PbI_2 in deuterated isopropanol.

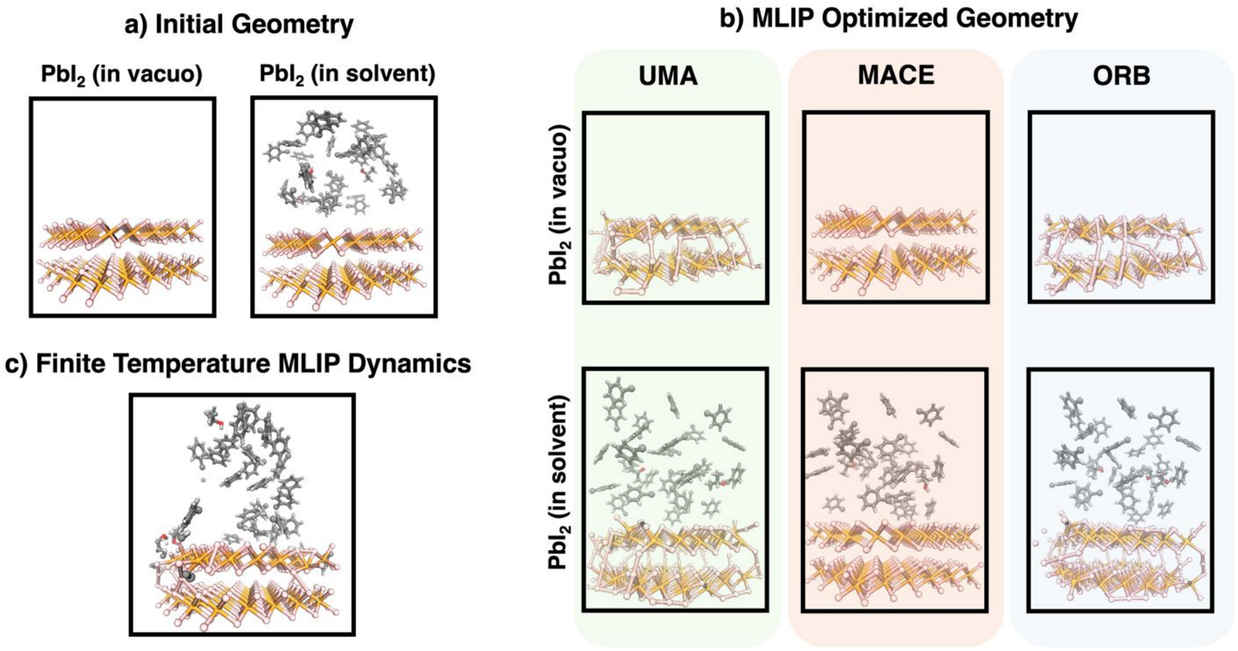

Supplementary Fig. 13 | surface chemistry examination of bidentate and monodentate surfaces using different MLIPs. 0K BFGS geometry optimization was performed with different MLIPs, and only MACE yielded the layered PbI_2 structure. All three MLIPs failed the finite-temperature MD simulation as the PbI_2 surface degraded very dramatically with only the solution on the surface.

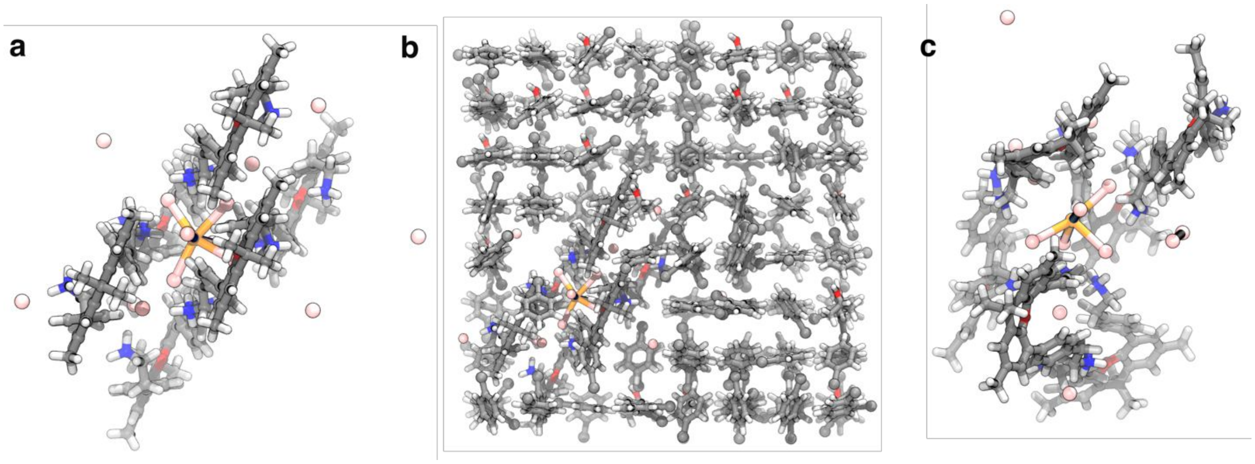

Supplementary Fig. 14 | Crystal seed construction and simulation. (a) The initial configuration of one example crystal seed (MeX-crystal-derived protocol). (b) Solvated crystal seed in chlorobenzene and isopropanol. (c) Unphysical structures in the simulation that persist for at least 30 ps. The additional Iodide ions for balancing the total charge of the system somehow "bonded" with another adjacent Iodide ion, leaving two charges removed from the system.

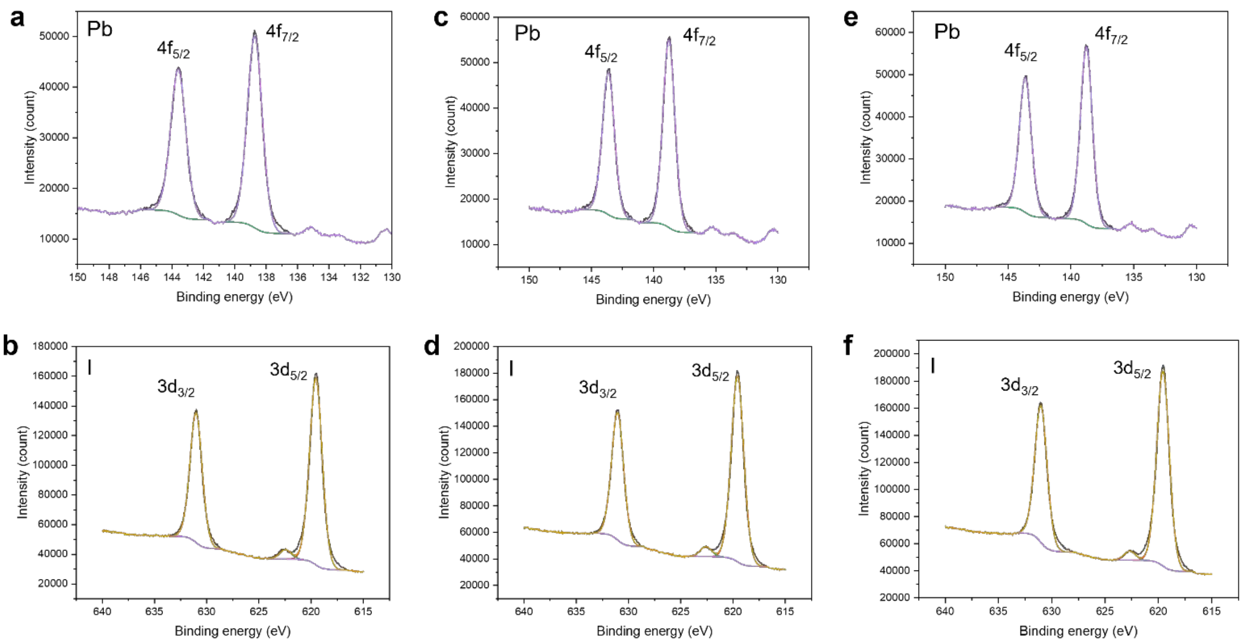

Supplementary Fig. 15 | High-resolution XPS of ligand-treated $FAPbI_3$ films**.** (a, c, and e), Pb 4f spectra of pristine $FAPbI_3$ (a), PEA-$FAPbI_3$ (c), and TEA-$FAPbI_3$ (e). (b, d, and f), corresponding I 3d spectra. Black lines are the fitted envelopes; colored curves denote individual components.

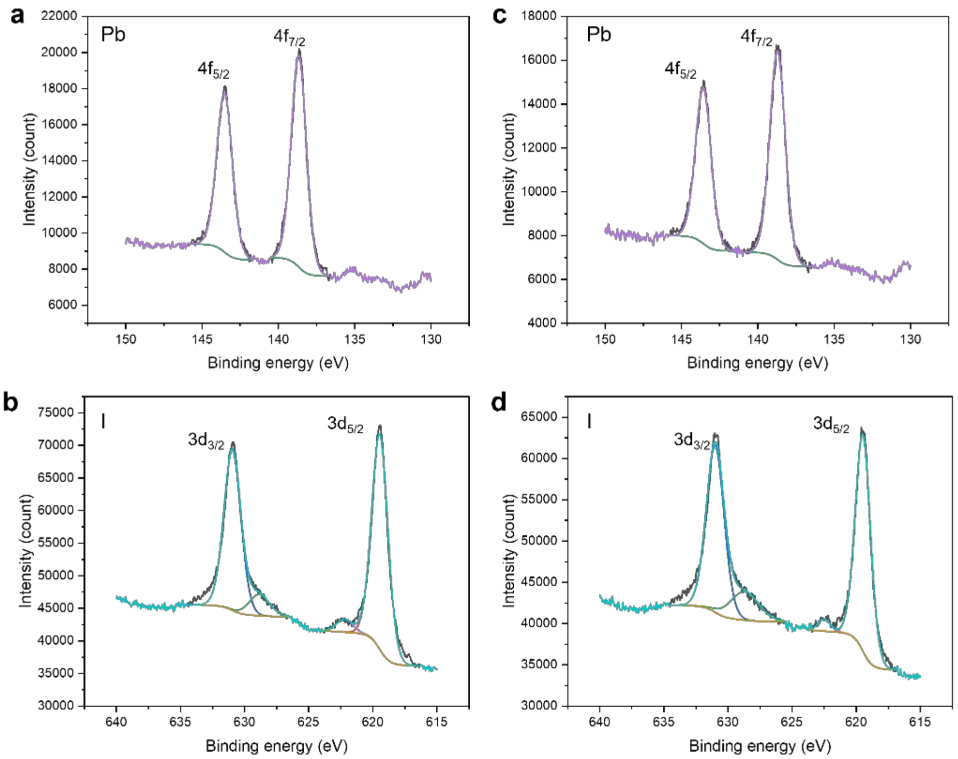

Supplementary Fig. 16 | High-resolution XPS of ligand-treated $FAPbI_3$ films**.** (a and c), Pb 4f spectra of MeX-$FAPbI_3$ (a) and MeXT-$FAPbI_3$ (c). b and d, corresponding I 3d spectra. Black lines are the fitted envelopes; colored curves denote individual components.

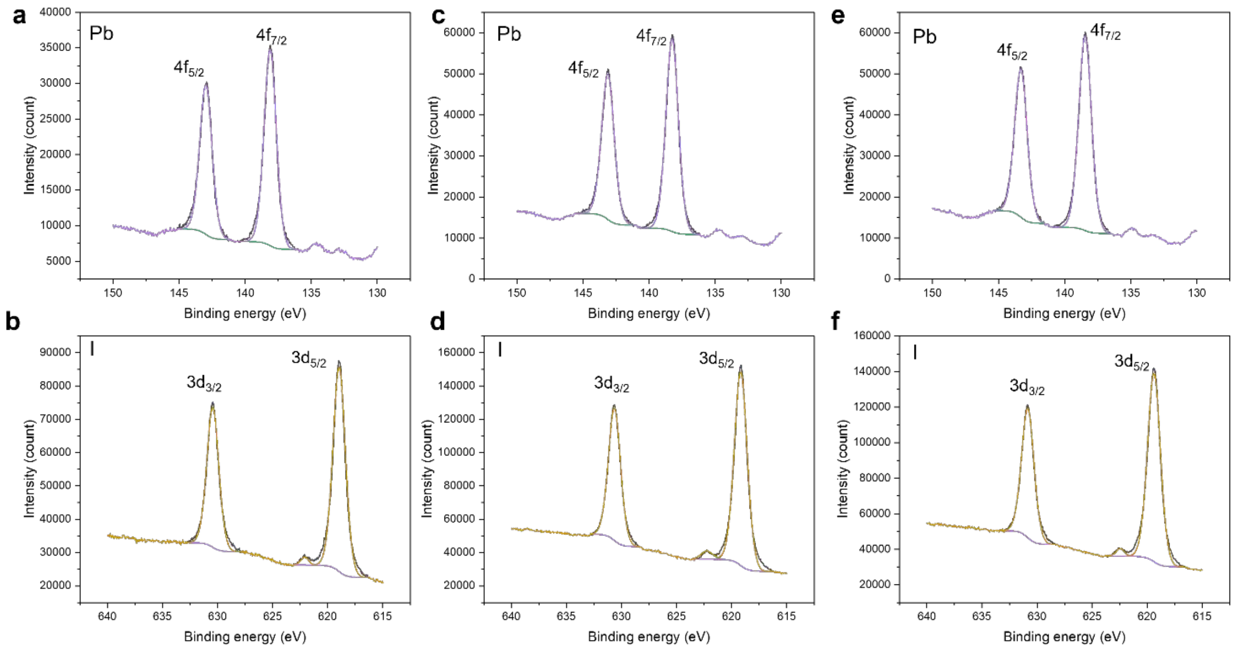

Supplementary Fig. 17 | High-resolution XPS of ligand-treated PbI_2 films**.** (a, c, and e), Pb 4f spectra of pristine PbI_2 (a), PEA-PbI_2 (c), and TEA-PbI_2 (e). (b, d, and f) corresponding I 3d spectra. Black lines are the fitted envelopes; colored curves denote individual components.

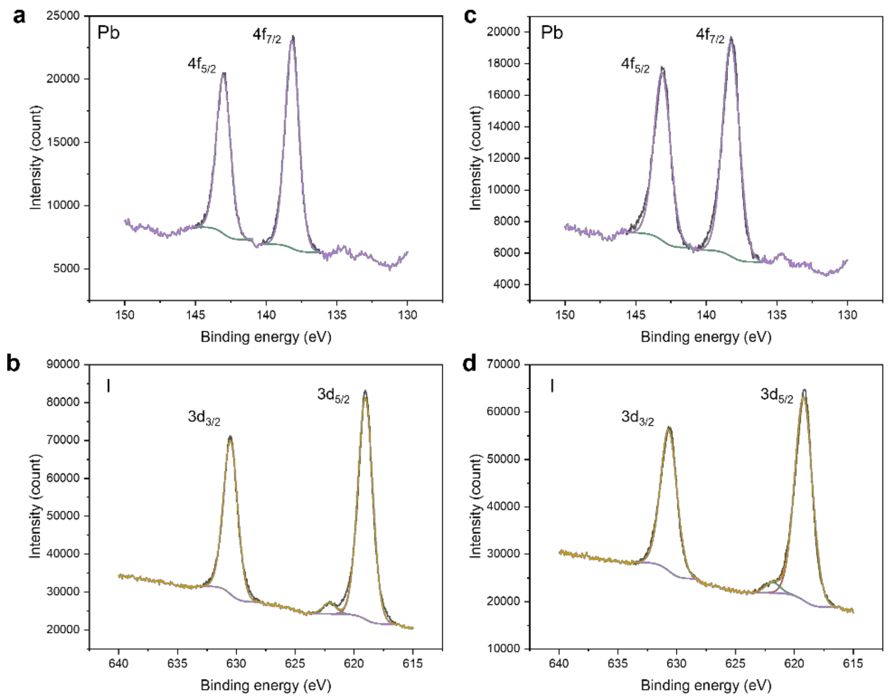

Supplementary Fig. 18 | High-resolution XPS of ligand-treated PbI_2 films**.** (a and c), Pb 4f spectra of MeX-PbI_2 (a) and MeXT-PbI_2 (c). (b and d), corresponding I 3d spectra. Black lines are the fitted envelopes; colored curves denote individual components.

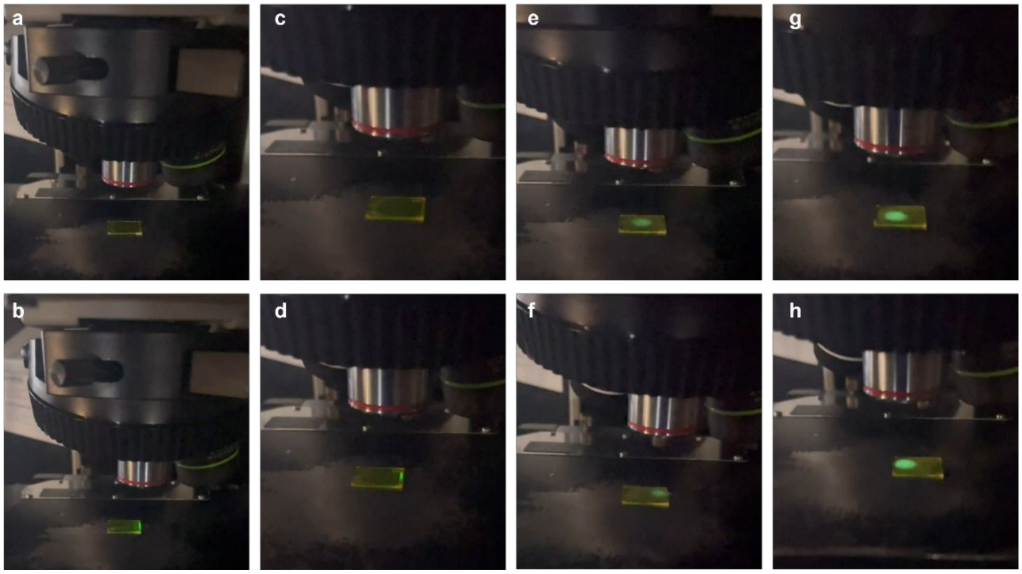

Supplementary Fig. 19 | Optical micrographs of ligand-treated PbI_2 films. Images taken at the film center (a, c, e, g) and edge (b, d, f, h) under 375 nm excitation for PEA-PbI_2 (a, b), TEA-PbI_2 (c, d), MeX-PbI_2 (e, f), and MeXT-PbI_2 (g, h).

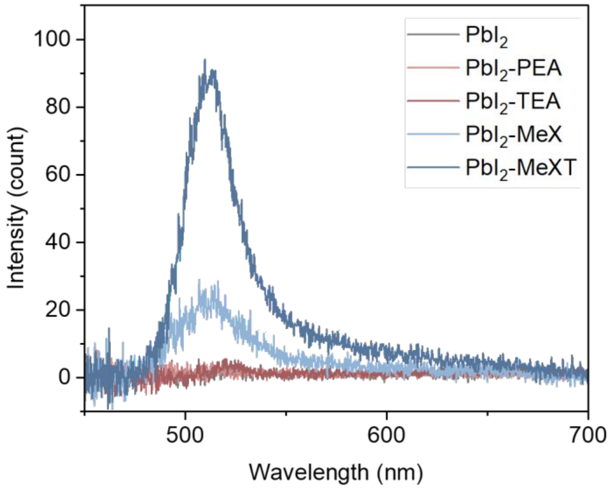

Supplementary Fig. 20 | PL intensity of the middle part of ligand-treated PbI_2 films.

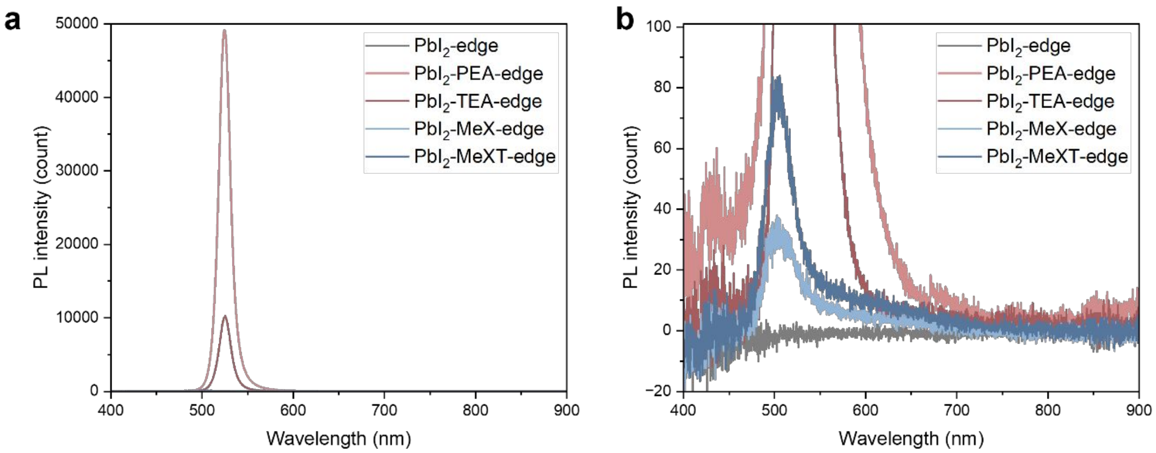

Supplementary Fig. 21 | (a) Steady-state PL spectra (λ_{exc}= 375 nm) measured at the film edge for pristine PbI_2 and samples treated with monodentate PEA and TEA or bidentate MeX and MeXT. (b), Same data on an expanded intensity scale to emphasize the weaker emission from MeX/MeXT-treated films. The sharp 520 nm peak originates from 2D perovskite.

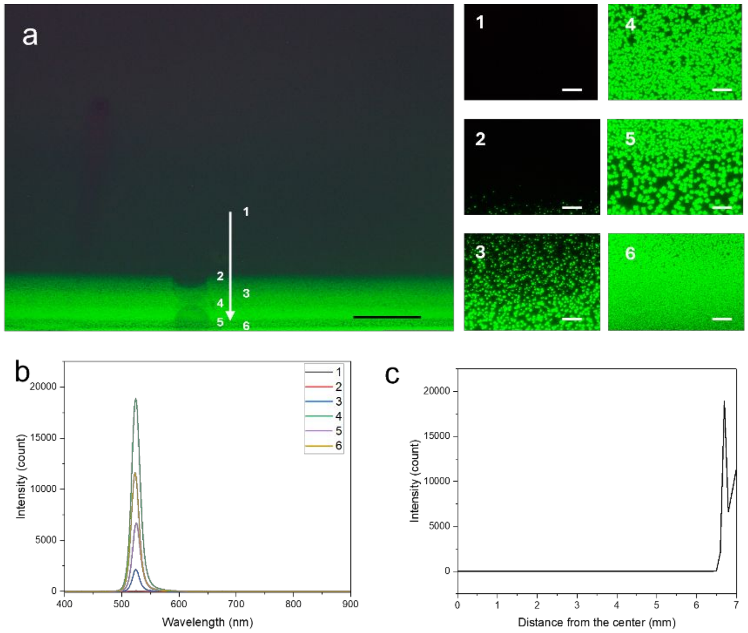

Supplementary Fig. 22 | Spatially resolved PL characterization of PbI_2 films treated with PEA. (a) Large-area PL mapping near the film edge (scale bar: 500 μm). Positions 1–6 are marked, and their corresponding zoomed-in PL maps are displayed on the right (scale bar: 20 μm). (b) Representative PL spectra extracted from positions 1–6. (c) PL intensity as a function of distance from the film center.

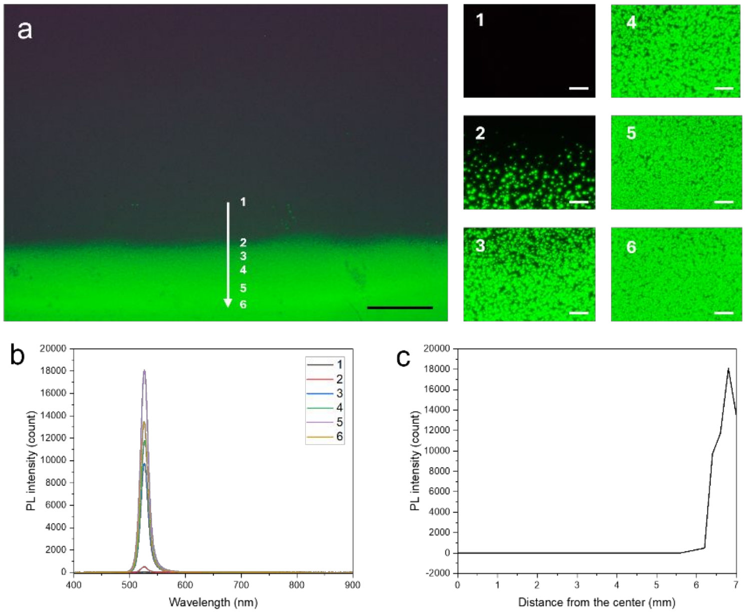

Supplementary Fig. 23 | Spatially resolved PL characterization of PbI_2 films treated with TEA. (a) Large-area PL mapping near the film edge (scale bar: 500 μm). Positions 1–6 are marked, and their corresponding zoomed-in PL maps are displayed on the right (scale bar: 20 μm). (b) Representative PL spectra extracted from positions 1–6. (c) PL intensity as a function of distance from the film center.

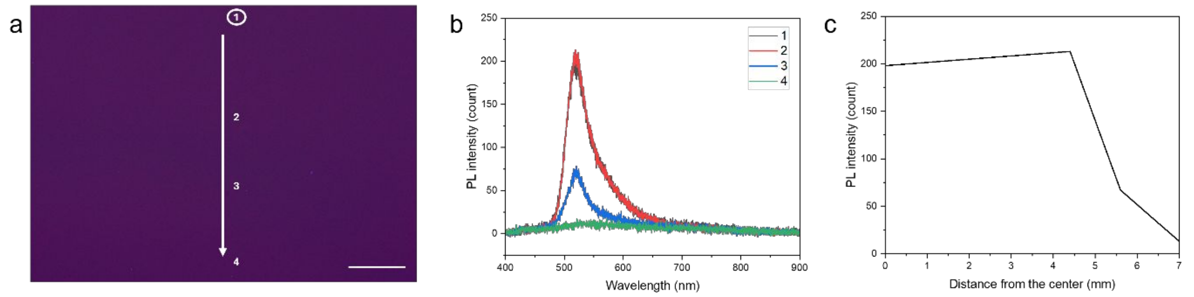

Supplementary Fig. 24 | Spatially resolved PL characterization of PbI_2 films treated with MeX. (a) Large-area PL mapping near the film edge (scale bar: 500 μm). (b) Representative PL spectra extracted from positions 1–4. (c) PL intensity as a function of distance from the film center. Position 1 (circled) lies outside the effective edge-gradient region and corresponds to a more central area of the film.

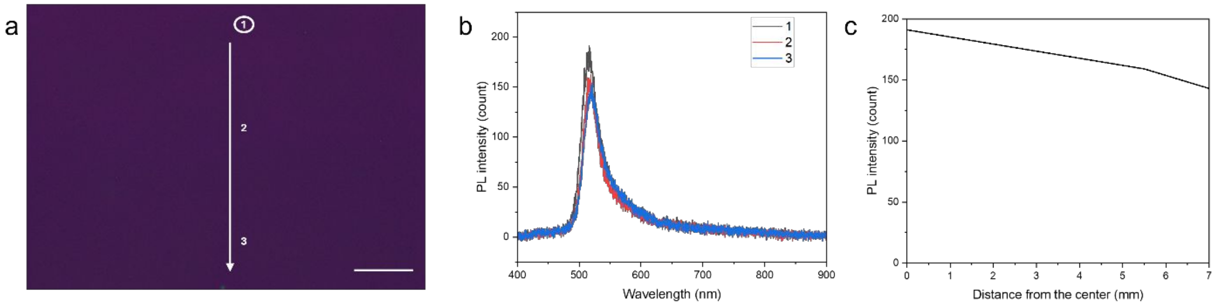

Supplementary Fig. 25 | Spatially resolved PL characterization of PbI_2 films treated with MeXT. (a) Large-area PL mapping near the film edge (scale bar: 500 μm). (b) Representative PL spectra extracted from positions 1–3. (c) PL intensity as a function of distance from the film center. Position 1 (circled) lies outside the effective edge-gradient region and corresponds to a more central area of the film.

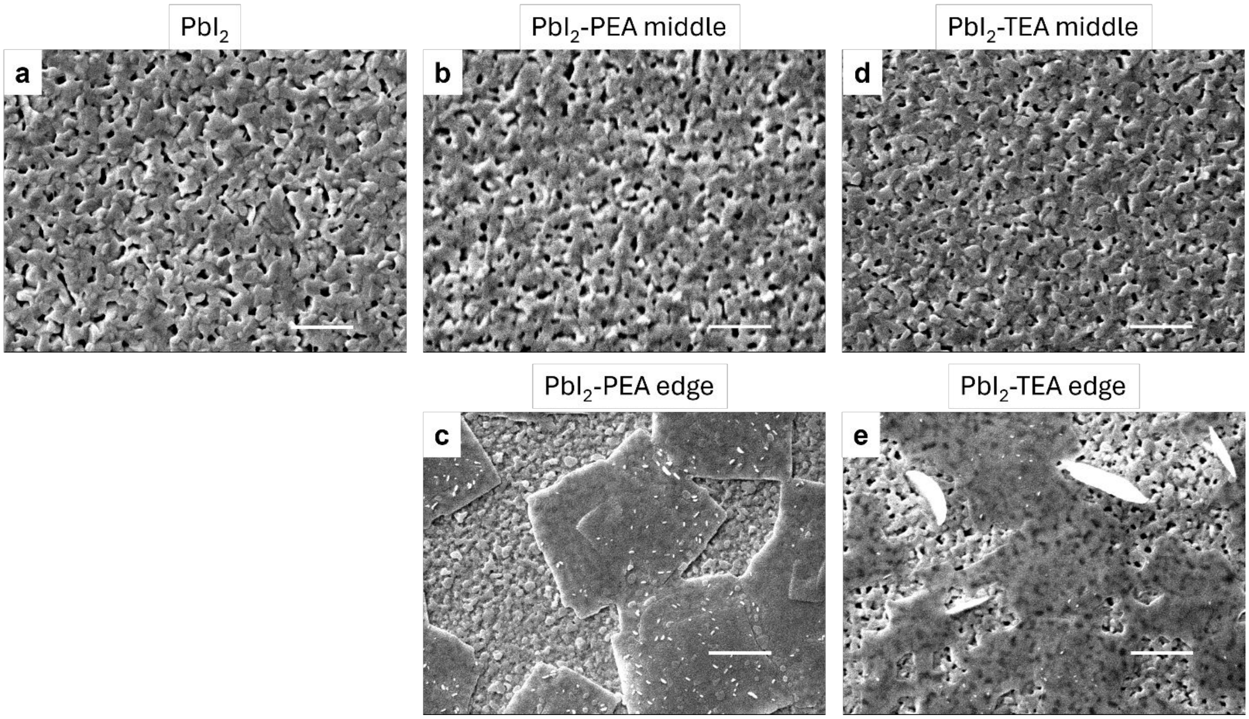

Supplementary Fig. 26 | (a) Pristine PbI_2 film. (b,c) PbI_2-PEA and (d,e) PbI_2-TEA films, showing central (b,d) and edge (c,e) regions (scale bar: 1 μm).

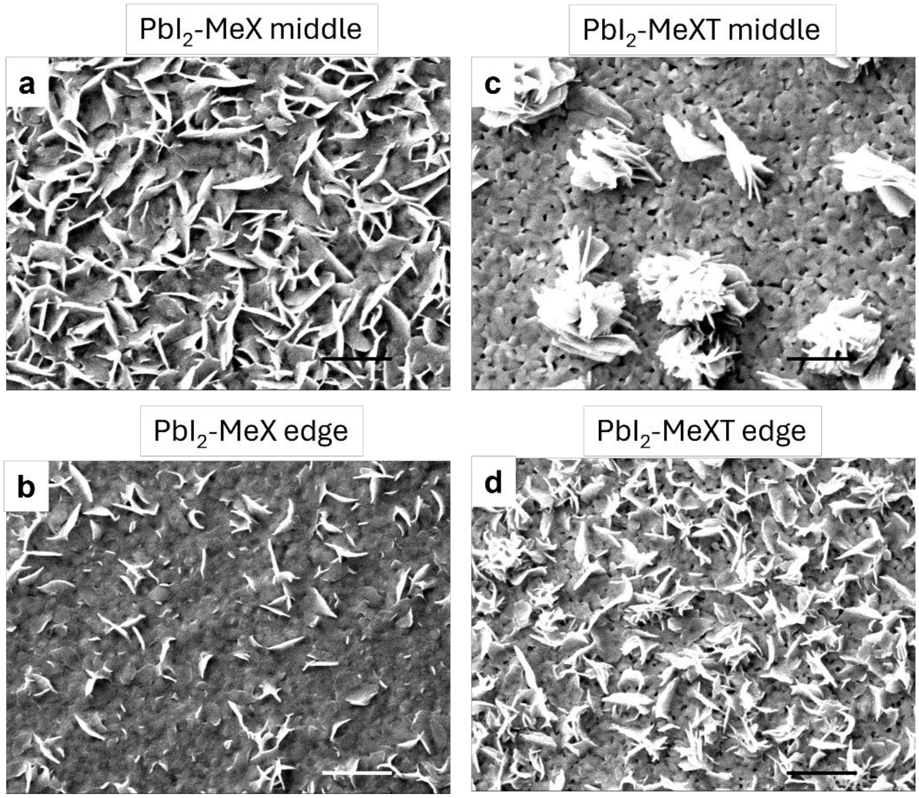

Supplementary Fig. 27 | (a,b) PbI_2-MeX and (c,d) PbI_2-MeXT films, showing representative morphologies at the central (a,c) and edge (b,d) regions of the films (scale bar: 1 μm).

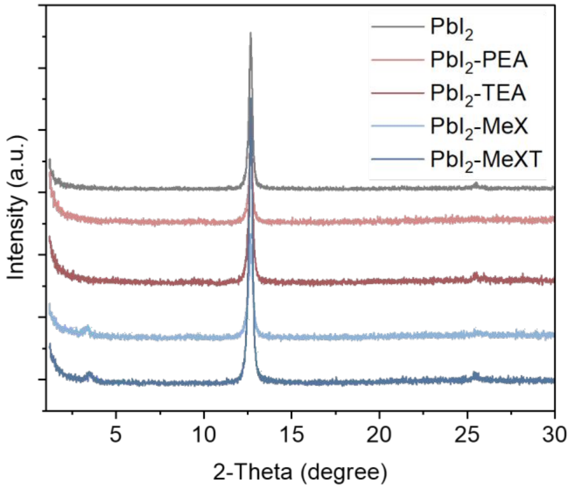

Supplementary Fig. 28 | XRD spectra of the middle part of ligand-treated PbI_2 films.

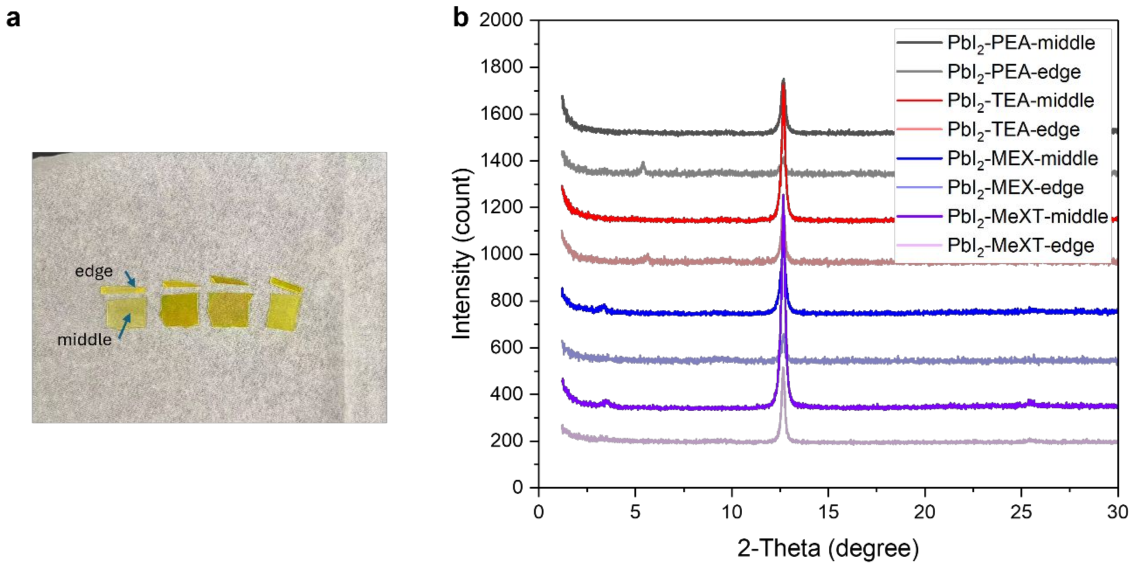

Supplementary Fig. 29 | (a) Photograph indicating the regions probed: “middle” corresponds to the film center, “edge” to the perimeter. b, XRD collected from those two positions for samples treated with monodentate PEA and TEA or bidentate MeX and MeXT.

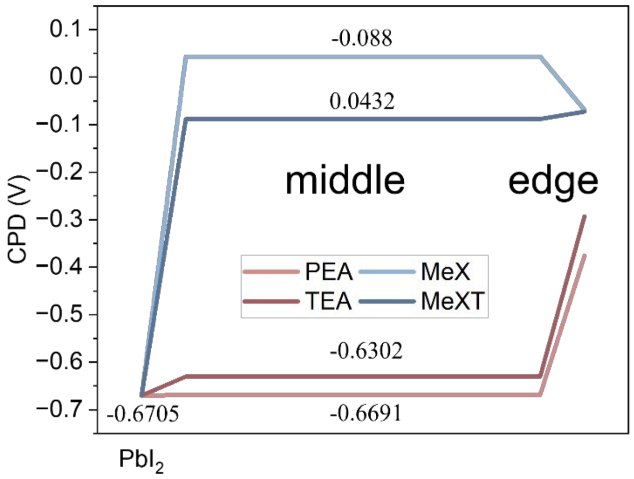

Supplementary Fig. 30 | CPD line profile from KPFM for ligand-treated PbI_2 films.

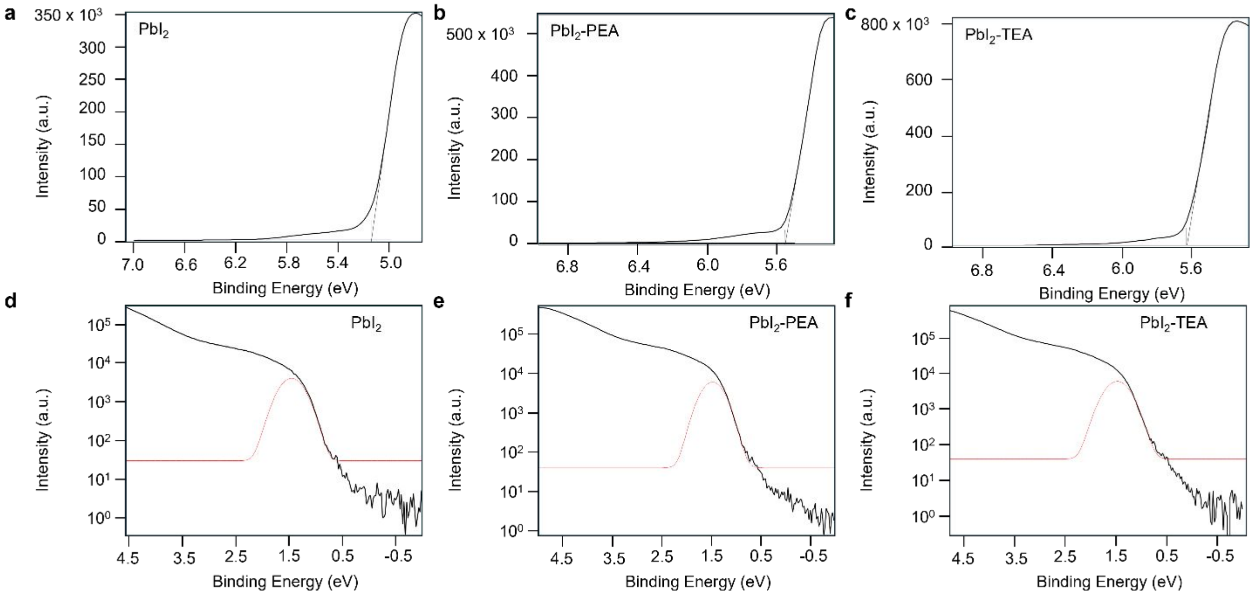

Supplementary Fig. 31 | (a–c), Secondary-electron cut-off spectra used to determine the work function of pristine PbI_2 (a), PEA-PbI_2 (b), and TEA-PbI_2 (c). d–f, Corresponding valence-band edge spectra; red lines are Gaussian fits used to extract the valence-band maximum following the procedure described by Endres, et al.[28] All spectra are plotted with respect to the Fermi level at a binding energy of 0 eV.

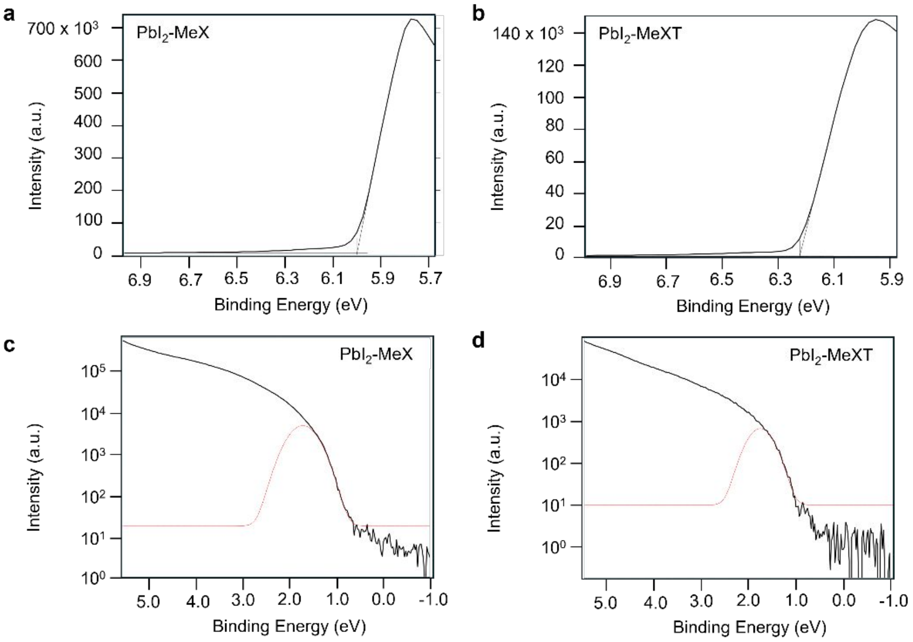

Supplementary Fig. 32 | (a, b), Secondary-electron cut-off spectra used to determine the work function of MeX-PbI_2 (a) and MeXT-PbI_2 (b). (c, d), Corresponding valence-band edge spectra; red lines are Gaussian fits used to extract the valence-band maximum following the procedure described by Endres et al.[28] All spectra are plotted with respect to the Fermi level at a binding energy of 0 eV.

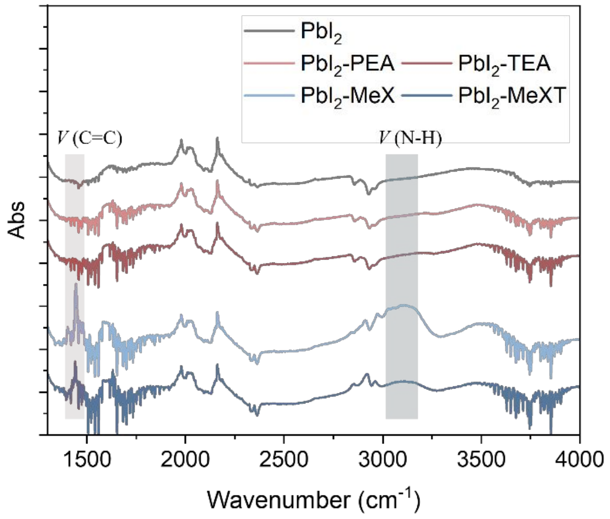

Supplementary Fig. 33 | FTIR of PbI_2 before and after ligand treatment. Shaded windows mark the aromatic *v*(C=C) region (≈1420-1450 cm^{-1}, left) and the ammonium *v*(N–H) stretching region (≈3000–3300 cm^{-1}, right).

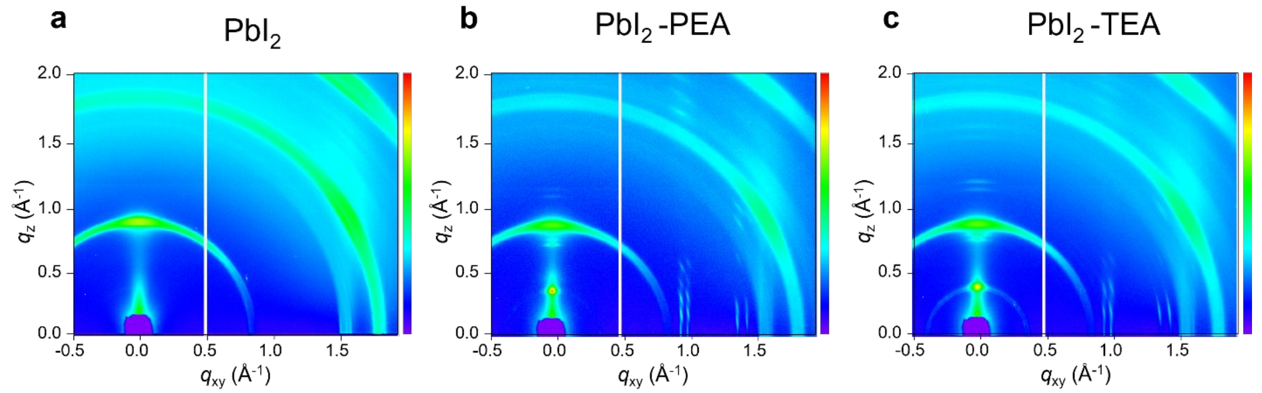

Supplementary Fig. 34 | GIWAX for pristine PbI_2 (a), PEA-PbI_2 (b), and TEA-PbI_2 (c). The intense (00l) arcs at $q_z \approx 0.45$ Å^{-1} indicate vertically oriented 2D phases that emerge after PEA and TEA treatment, whereas the untreated film shows only the broad PbI_2 halo.

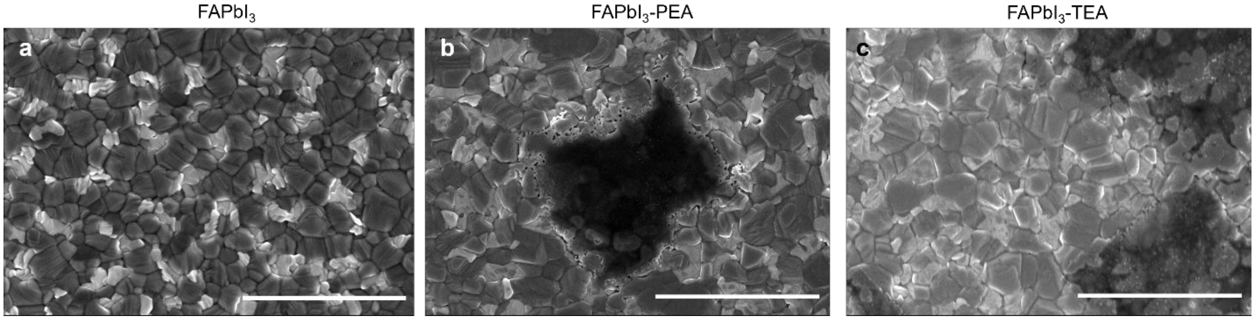

Supplementary Fig. 35 | Top-view SEM of monodentate-treated $FAPbI_3$ films. (a), Untreated $FAPbI_3$ exhibits compact, faceted grains. (b, c), Post-treatment with PEA (b) or TEA (c) exhibits localized large 2D nanosheets, while the surrounding white PbI_2 remains largely intact. Scale bars, 5 μm.

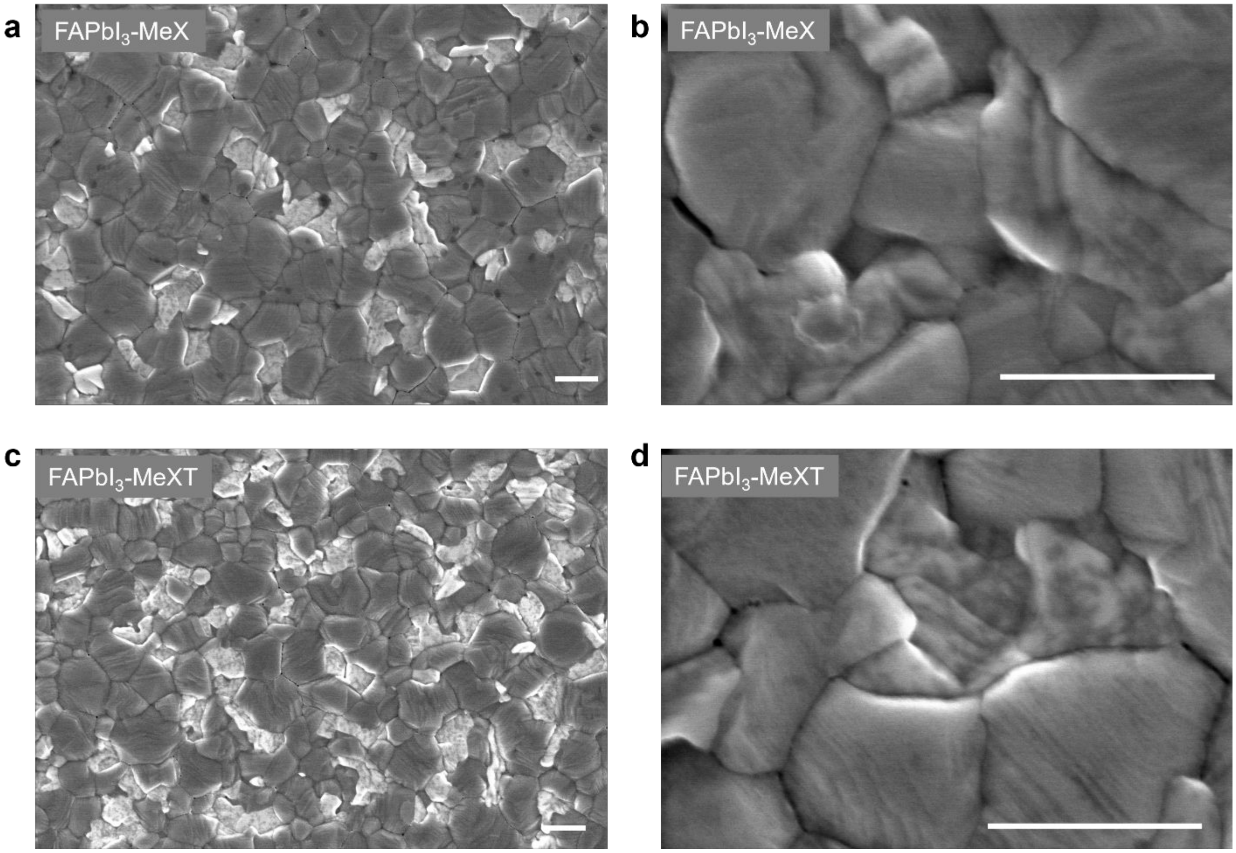

Supplementary Fig. 36 | Top-view SEM of bidentate-treated $FAPbI_3$ films. (a, c), Low-magnification images of MeX- (a) and MeXT- (c) passivated $FAPbI_3$ show passivated PbI_2 grains comparable to the pristine film. (b, d), Higher-magnification views reveal a clear passivated PbI_2 surface. Scale bars, 1 μm.

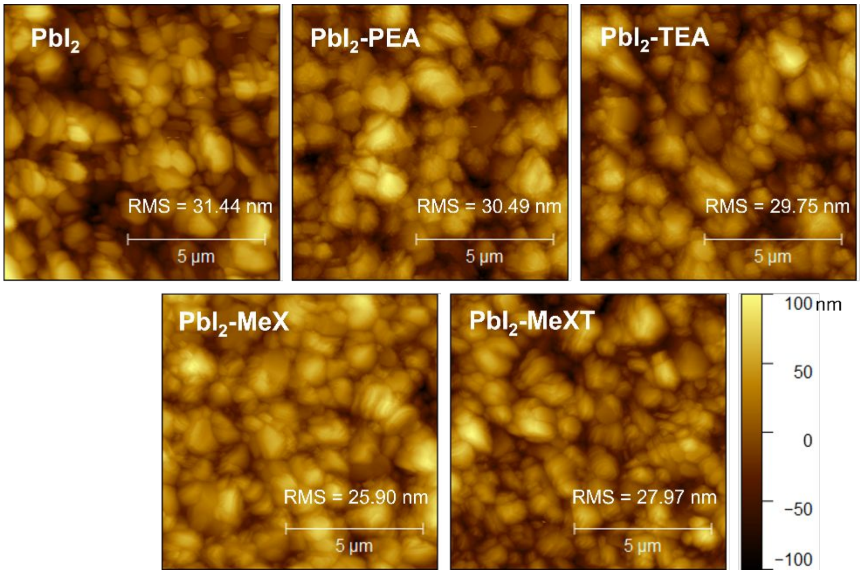

Supplementary Fig. 37 | AFM height images of pristine PbI_2 and films treated with PEA, TEA, MeX, and MeXT. RMS: root-mean-square roughness.

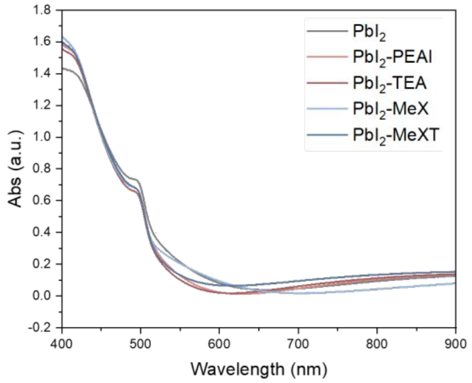

Supplementary Fig. 38 | Absorption spectra of pristine PbI_2 and films treated with PEA, TEA, MeX, and MeXT (scale bar: 1 μm).

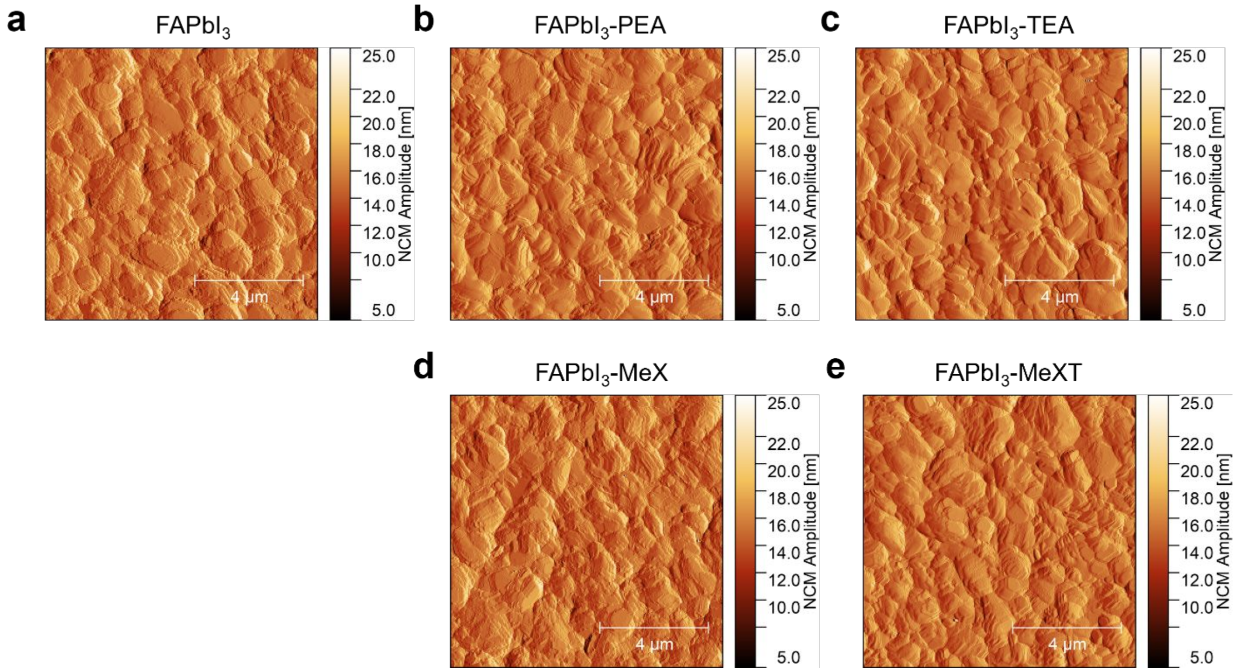

Supplementary Fig. 39 | (a–e), Non-contact amplitude images (10 μm × 10 μm) for the pristine $FAPbI_3$ film (a) and after post-treatment with PEA (b), TEA (c), MeX (d), and MeXT (e). All samples maintain a dense, terraced grain structure. Scale bars, 4 μm.

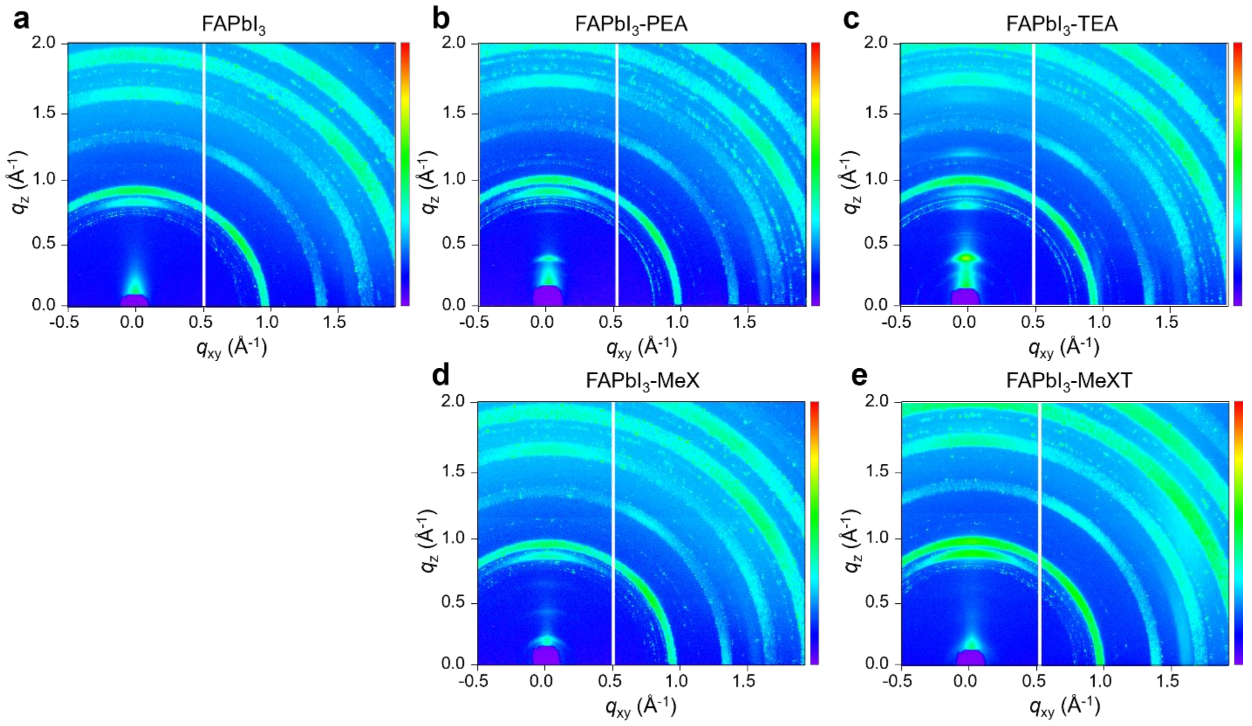

Supplementary Fig. 40 | GIWAX for pristine $FAPbI_3$ (a) and films treated with PEA (b), TEA (c), MeX (d), and MeXT (e). Monodentate ligands induce faint anisotropic streaks for 2D RP formation, whereas bidentate MeX/MeXT shows relatively isotropic ring patterns of the 2D phase.

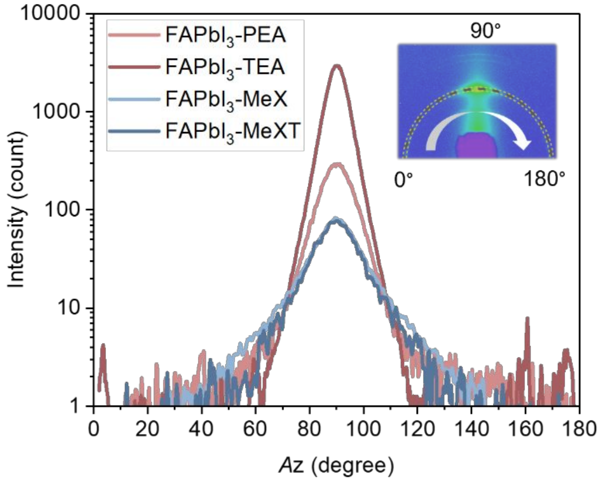

Supplementary Fig. 41 | Azimuthal intensity profiles of the (001) diffraction ring associated with the 2D perovskite formed on $FAPbI_3$ films after passivation with PEA, TEA, MeX, and MeXT, respectively.

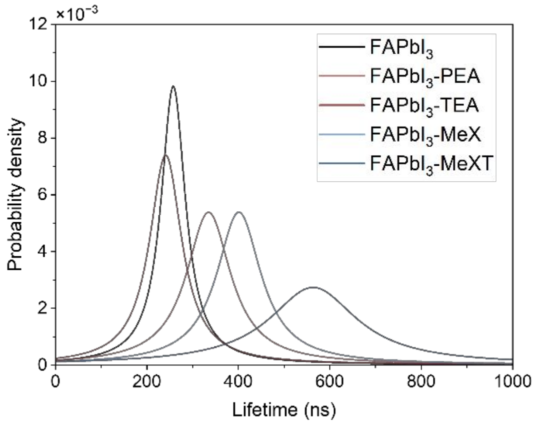

Supplementary Fig. 42 | Probability density distributions of the longest lifetime component (τ_3) extracted from multi-exponential fitting of FLIM decay traces for $FAPbI_3$ films with different ligand treatments. The distributions are constructed from the τ_3 values across the mapped area and presented as smoothed probability density curves.

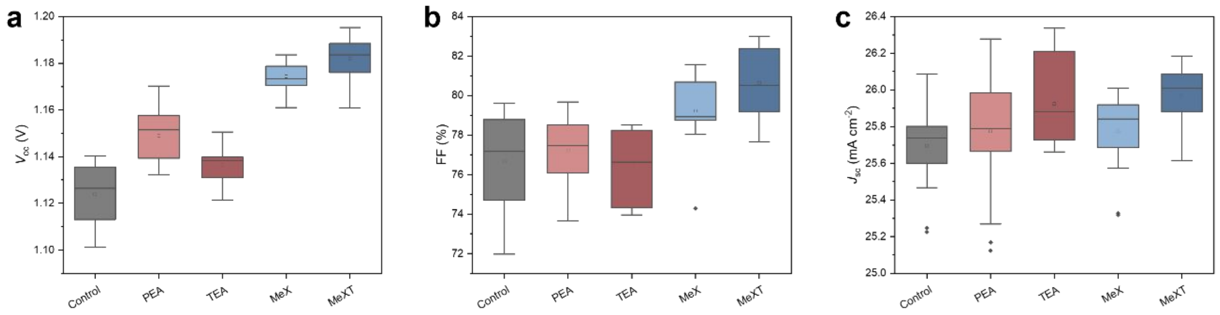

Supplementary Fig. 43 | Box-and-whisker plots comparing V_{oc} (a), FF (b), and J_{sc} (c) for control, PEA-, TEA-, MeX-, and MeXT-passivated devices. Boxes span the interquartile range, whiskers mark the full range, central lines show medians, and squares denote means. All boxes display the mean value, with 1.5× outlier range whiskers.

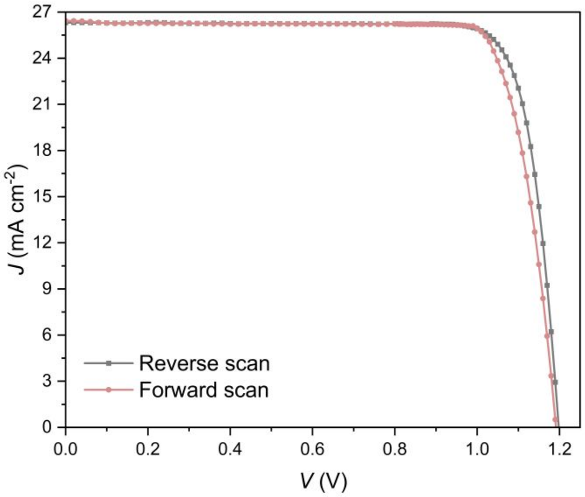

Supplementary Fig. 44 | Forward and reverse scans for champion MeXT-passivated device.

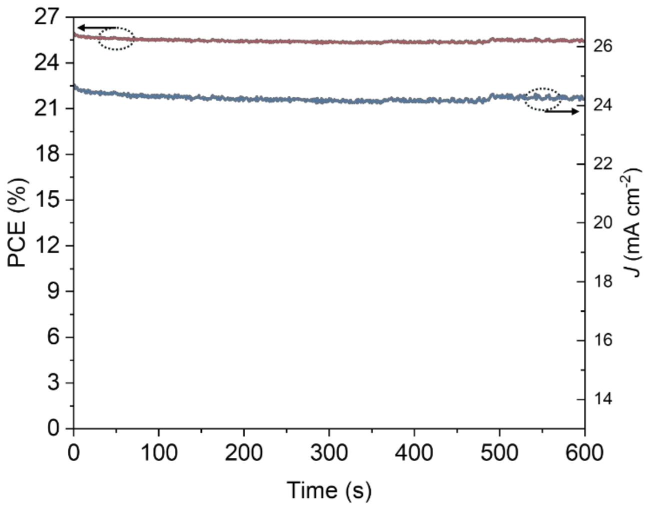

Supplementary Fig. 45 | Maximum-power-point (MPP) tracking of the champion MeXT-passivated device. Stabilized power conversion efficiency (left axis) and photocurrent density (right axis) under continuous 1-sun illumination at 45 °C for 10 min in a glovebox. The device settles at 25.65 % after an initial 20 s burn-in.

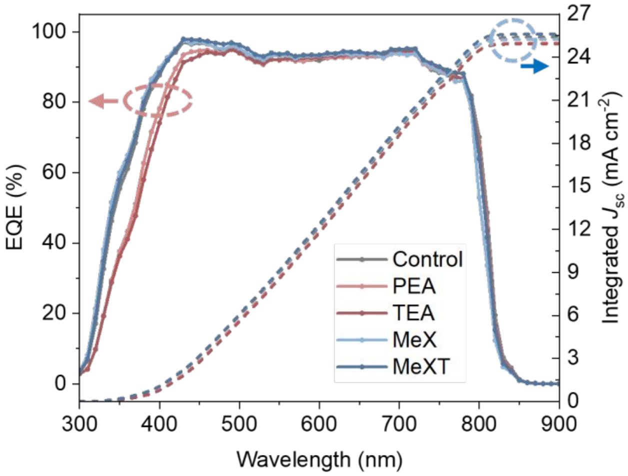

Supplementary Fig. 46 | EQE plots and integrated *J*sc values of the devices with different ligand passivation.

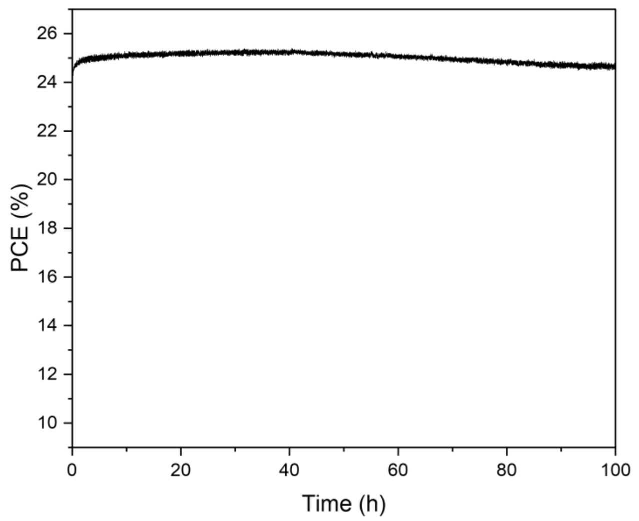

Supplementary Fig. 47 | 100-h operational stability of the MeXT-passivated device without an anti-reflection layer. An unencapsulated cell held at MPP under 1-sun, 45 °C illumination retains > 95 % of its initial efficiency after 100 h, demonstrating excellent short-term operational robustness.

Supplementary Table 1 | Crystal data and structure refinement for (MeXT)$PbBr_4$ with structural disorder (150 K).

CCDC number	2548865
Empirical formula	$C_{58.01}H_{68.01}Br_{7.41}N_4O_2Pb_{1.71}S_4$
Formula weight	1927.12
Temperature/K	150(2)
Crystal system	monoclinic
Space group	$P2_1/n$
a/Å	16.118(8)
b/Å	8.610(5)
c/Å	56.806(15)
α/°	90
β/°	92.65(3)
γ/°	90
Volume/Å^3	7875(6)
Z	4
ρ_{calc}g/cm^3	1.625
μ/mm^{-1}	7.545
F(000)	3693.0
Crystal size/mm^3	0.120 × 0.110 × 0.070
Radiation	MoKα (λ = 0.71073)
2Θ range for data collection/°	4.294 to 66.338
Index ranges	-24 ≤ h ≤ 24, -13 ≤ k ≤ 13, -87 ≤ l ≤ 87
Reflections collected	224989
Independent reflections	29952 [R_{int} = 0.0745, R_{sigma} = 0.0519]
Data/restraints/parameters	29952/4908/1542
Goodness-of-fit on F^2	1.016
Final R indexes [I>=2σ (I)]	R_1 = 0.0677, wR_2 = 0.1744
Final R indexes [all data]	R_1 = 0.1216, wR_2 = 0.2009
Largest diff. peak/hole / e Å^{-3}	1.71/-2.00

Supplementary Table 2 | XPS peak areas, RSFs, and calculated Pb / I atomic ratios for pristine and ligand-treated $FAPbI_3$ and PbI_2 films.

	**Pb 4f7/2 Area**	**Pb 4f7/2 R.S.F.**	**Pb 4f5/2 Area**	**Pb 4f5/2 R.S.F.**	**I 3f5/2 Area**	**I 3f5/2 R.S.F.**	**I 3f3/2 Area**	**I 3f3/2 R.S.F.**	**I sate Area**	**I sate R.S.F**	**Pb/I ratio**
**$FAPbI_3$**	20672	0.285	15830	0.223	46976	0.429	31085	0.296			**0.669**
**$FAPbI_3$ -PEA**	22791	0.285	17311	0.223	52119	0.429	33902	0.296			**0.668**
**$FAPbI_3$ -TEA**	22839	0.285	17641	0.223	52771	0.429	35691	0.296	2124	1	**0.648**
**$FAPbI_3$ -MeX**	6605	0.285	5105	0.223	12769	0.429	9926	0.296	2347	1	**0.702**
**$FAPbI_3$ -MeXT**	5495	0.285	4123	0.223	9626	0.429	8564	0.296	2736	1	**0.698**
**PbI_2**	14565	0.285	11152	0.223	21544	0.429	13949	0.296	487	1	**1.033**
**PbI_2-PEA**	25354	0.285	19251	0.223	41946	0.429	26856	0.296	1596	1	**0.922**
**PbI_2-TEA**	26228	0.285	19664	0.223	38393	0.429	25187	0.296	893	1	**1.027**
**PbI_2-MeX**	9717	0.285	7090	0.223	22570	0.429	14987	0.296	1007	1	**0.632**
**PbI_2-MeXT**	9162	0.285	7043	0.223	19553	0.429	12627	0.296	860	1	**0.715**

Supplementary Table 3 | UPS-derived work function (WF), valence-band maximum (VBM) onset, and ionization energy (IE) for pristine and ligand-treated PbI_2 films.

	WF (eV)	HOMO/VBM onset (eV)	IE/HOMO/VBM (eV)
**PbI_2**	5.05	0.83	5.88
**PbI_2-PEA**	4.65	0.85	5.50
**PbI_2-TEA**	4.60	0.81	5.41
**PbI_2-MeX**	4.20	0.90	5.10
**PbI_2-MeXT**	3.95	1.05	5.00

Supplementary Table 4 | Tri-exponential TRPL fit parameters and intensity-weighted average lifetime (τ_{avg}) of pristine and ligand-treated $FAPbI_3$ films.

	A1	τ_1 (ns)	A2	τ_2(ns)	A3	τ_3(ns)	τ_{avg}(ns)
**$FAPbI_3$**	0.471	1.345	0.190	39.334	0.289	222.252	201.420
**$FAPbI_3$-PEA**	0.506	1.262	0.174	91.009	0.278	380.279	340.810
**$FAPbI_3$-TEA**	0.450	1.215	0.252	44.105	0.269	316.624	283.583
**$FAPbI_3$-MeX**	0.452	1.631	0.182	81.286	0.258	369.130	328.240
**$FAPbI_3$-MeXT**	0.480	1.665	0.182	118.601	0.258	493.298	436.629

Reference

1 Lin, C. J. *et al.* Intralayer bidentate diammoniums for stable two-dimensional perovskites. *Nature Chemistry* (2026). https://doi.org/10.1038/s41557-025-02038-w

2 Yin, J. W., Khalilov, A. N., Muthupandi, P., Ladd, R. & Birman, V. B. Phenazine-1,6-dicarboxamides: Redox-Responsive Molecular Switches. *Journal of the American Chemical Society* **142**, 60–63 (2020). https://doi.org/10.1021/jacs.9b11160

3 Wei, Z. T. *et al.* A selenophene-containing conjugated organic ligand for two-dimensional halide perovskites. *Chemical Communications* **57**, 11469–11472 (2021). https://doi.org/10.1039/d1cc04679a

4 Gaussian 16 Rev. B.01 (Wallingford, CT, 2016).

5 Chai, J. D. & Head-Gordon, M. Long-range corrected hybrid density functionals with damped atom-atom dispersion corrections. *Physical Chemistry Chemical Physics* **10**, 6615–6620 (2008). https://doi.org/10.1039/b810189b

6 Krishnan, R., Binkley, J. S., Seeger, R. & Pople, J. A. Self-Consistent Molecular-Orbital Methods .20. Basis Set for Correlated Wave-Functions. *Journal of Chemical Physics* **72**, 650–654 (1980). https://doi.org/10.1063/1.438955

7 Humphrey, W., Dalke, A. & Schulten, K. VMD: Visual molecular dynamics. *Journal of Molecular Graphics & Modelling* **14**, 33–38 (1996). https://doi.org/10.1016/0263-7855(96)00018-5

8 Kresse, G. Ab-Initio Molecular-Dynamics for Liquid-Metals. *Journal of Non-Crystalline Solids* **193**, 222–229 (1995). https://doi.org/Doi 10.1016/0022-3093(95)00355-X

9 Kresse, G. & Furthmuller, J. Efficiency of ab-initio total energy calculations for metals and semiconductors using a plane-wave basis set. *Computational Materials Science* **6**, 15–50 (1996). https://doi.org/10.1016/0927-0256(96)00008-0

10 Kresse, G. & Furthmuller, J. Efficient iterative schemes for ab initio total-energy calculations using a plane-wave basis set. *Physical Review B* **54**, 11169–11186 (1996). https://doi.org/10.1103/PhysRevB.54.11169

11 Kresse, G. & Joubert, D. From ultrasoft pseudopotentials to the projector augmented-wave method. *Physical Review B* **59**, 1758–1775 (1999). https://doi.org/10.1103/PhysRevB.59.1758

12 Perdew, J. P., Burke, K. & Ernzerhof, M. Generalized gradient approximation made simple. *Physical Review Letters* **77**, 3865–3868 (1996). https://doi.org/10.1103/PhysRevLett.77.3865

13 Grimme, S., Antony, J., Ehrlich, S. & Krieg, H. A consistent and accurate ab initio parametrization of density functional dispersion correction (DFT-D) for the 94 elements H-Pu. *Journal of Chemical Physics* **132** (2010). https://doi.org/10.1063/1.3382344

14 Minagawa, T. Five new polytypes and polytypic change in PbI2. *Journal of Applied Crystallography* **12**, 57–59 (1979). https://doi.org/10.1107/S0021889879011778

15 Weller, M. T., Weber, O. J., Frost, J. M. & Walsh, A. Cubic Perovskite Structure of Black Formamidinium Lead Iodide, α-[HC(NH)]PbI3, at 298 K. *Journal of Physical Chemistry Letters* **6**, 3209–3212 (2015). https://doi.org/10.1021/acs.jpclett.5b01432
16 Grimme, S., Bannwarth, C. & Shushkov, P. A Robust and Accurate Tight-Binding Quantum Chemical Method for Structures, Vibrational Frequencies, and Noncovalent Interactions of Large Molecular Systems Parametrized for All spd-Block Elements (=1-86). *Journal of Chemical Theory and Computation* **13**, 1989–2009 (2017). https://doi.org/10.1021/acs.jctc.7b00118
17 Kühne, T. D. *et al.* CP2K: An electronic structure and molecular dynamics software package - Quickstep: Efficient and accurate electronic structure calculations. *Journal of Chemical Physics* **152** (2020). https://doi.org/10.1063/5.0007045
18 Batatia, I., Kovács, D. P., Simm, G. N. C., Ortner, C. & Csányi, G. MACE: Higher Order Equivariant Message Passing Neural Networks for Fast and Accurate Force Fields. *Advances in Neural Information Processing Systems 35, Neurips 2022* (2022).
19 Brandon M. Wood, M. D., Xiang Fu, Meng Gao, Muhammed Shuaibi, Luis Barroso-Luque, Kareem Abdelmaqsoud, Vahe Gharakhanyan, John R. Kitchin, Daniel S. Levine, Kyle Michel, Anuroop Sriram, Taco Cohen, Abhishek Das, Ammar Rizvi, Sushree Jagriti Sahoo, Zachary W. Ulissi, C. Lawrence Zitnick. UMA: A Family of Universal Models for Atoms. *arxiv* (2025). https://doi.org/10.48550/arxiv.2506.23971
20 Benjamin Rhodes, S. V., Vaidotas Šimkus, James Gin, Jonathan Godwin, Tim Duignan, Mark Neumann. Orb-v3: atomistic simulation at scale. *arxiv* (2025). https://doi.org/10.48550/arxiv.2504.06231
21 Larsen, A. H. *et al.* The atomic simulation environment-a Python library for working with atoms. *Journal of Physics-Condensed Matter* **29** (2017). https://doi.org/10.1088/1361-648X/aa680e
22 Ziegler, J. F. & Biersack, J. P. in *Treatise on Heavy-Ion Science: Volume 6: Astrophysics, Chemistry, and Condensed Matter* (ed D. Allan Bromley) 93–129 (Springer US, 1985).
23 Deng, B. W. *et al.* CHGNet as a pretrained universal neural network potential for charge-informed atomistic modelling. *Nature Machine Intelligence* **5**, 1031–1041 (2023). https://doi.org/10.1038/s42256-023-00716-3
24 Luis Barroso-Luque, M. S., Xiang Fu, Brandon M. Wood, Misko Dzamba, Meng Gao, Ammar Rizvi, C. Lawrence Zitnick, Zachary W. Ulissi. Open Materials 2024 (OMat24) Inorganic Materials Dataset and Models. *arxiv* (2024). https://doi.org/10.48550/arxiv.2410.12771
25 Ghahremanpour, M. M., van Maaren, P. J. & van der Spoel, D. The Alexandria library, a quantum-chemical database of molecular properties for force field development. *Scientific Data* **5** (2018). https://doi.org/10.1038/sdata.2018.62
26 Du, K. Z. *et al.* Two-Dimensional Lead(II) Halide-Based Hybrid Perovskites Templated by Acene Alkylamines: Crystal Structures, Optical Properties, and Piezoelectricity.

Inorganic Chemistry **56**, 9291–9302 (2017). https://doi.org/10.1021/acs.inorgchem.7b01094

27 Dammak, H., Elleuch, S., Feki, H. & Abid, Y. Synthesis, crystal structure, vibrational spectra, optical properties and theoretical investigation of a two-dimensional self-assembled organic-inorganic hybrid material. *Solid State Sciences* **61**, 1–8 (2016). https://doi.org/10.1016/j.solidstatesciences.2016.08.014

28 Endres, J. *et al.* Valence and Conduction Band Densities of States of Metal Halide Perovskites: A Combined Experimental-Theoretical Study. *Journal of Physical Chemistry Letters* **7**, 2722–2729 (2016). https://doi.org/10.1021/acs.jpclett.6b00946
